# Maternal diet shapes neonatal microbiome ontogenesis and neurometabolic resilience

**DOI:** 10.1080/19490976.2026.2684074

**Published:** 2026-06-09

**Authors:** Saurabh Kadyan, Gwoncheol Park, Leila Khalili, Cole Patoine, Moses Mayonu, Bo Wang, Gloria Salazar, Yuichiro Yamashiro, Ravinder Nagpal

**Affiliations:** a The Gut Biome Lab, Florida State University, Tallahassee, FL, USA; b Department of Health, Nutrition, and Food Sciences, Florida State University, Tallahassee, FL, USA; c Dairy Microbiology Division, ICAR-National Dairy Research Institute, Karnal, Haryana, India; d College of Engineering and Science: Chemistry and Chemical Engineering, Florida Institute of Technology, Melbourne, FL, USA; e Probiotics Research Laboratory, Juntendo University School of Medicine, Tokyo, Japan; f Center for Integrative Nutrition and Food Research, Florida State University, Tallahassee, FL, USA

**Keywords:** Developmental origins of health and disease, gut–brain axis, omega-3 fatty acids, neuroinflammation, synaptic plasticity

## Abstract

Maternal diet high in saturated fatty acids (SFA) promote infant gut dysbiosis and impairs metabolic and neurocognitive outcomes; however, the protective potential of maternal polyunsaturated fatty acids (PUFA), particularly omega-3 (n3), remains unclear. This study examined how maternal diets enriched in SFA (20% milk fat), omega-6 (n6; 20% corn oil), or omega-3 (n3; 19% olive oil + 1% fish oil) influence neonatal metabolism, neurodevelopment, the gut microbiome, the gut–blood–brain metabolomes, and the brain lipidome in C57BL/6 mice. The offspring were exposed to these diets only during gestation and lactation and then maintained on a Western-style diet for 10 weeks. Compared to SFA, maternal PUFA-rich diets induced distinct and persistent microbiome signatures and reshaped the gut and systemic metabolomic profiles into adulthood. The offspring of n3-fed dams displayed higher lean-to-fat mass ratios, improved ileal morphology, and enhanced gut epithelial integrity. Chronic low-grade inflammation (MCP-1) along the gut-blood-brain axis was markedly reduced in n3 offspring. Moreover, maternal n3 intake enhanced synaptic plasticity, suppressed neuroinflammation, and enriched brain lipids and metabolites associated with membrane integrity, neuronal signaling, and anti-inflammatory pathways. Overall, maternal omega-3 intake confers long-term neuroprotective effects by modulating brain lipid remodeling and the gut–brain–immune axis.

## Graphical abstract

**Figure:**
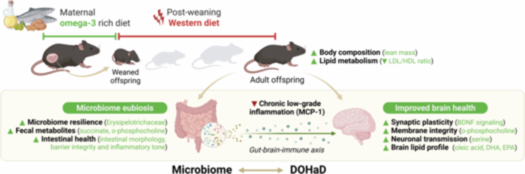

## Introduction

Early-life development represents a critical window in human lifespan, during which nutritional exposures strongly influence long-term health outcomes.^1^ The Developmental Origins of Health and Disease (DOHaD) theory posits that environmentally induced epigenetic changes during periods of growth, metabolism, and neurodevelopment shape lifelong susceptibility to chronic disorders.^2^ In particular, inadequate maternal and infant nutrition during the first 1000 d has been consistently associated with elevated risk of metabolic syndrome in later life.^3^ ^,^ ^4^ Accordingly, maternal adherence to nutrient-dense, health-promoting diets is a cornerstone for healthy infant development and the lifelong aging process.^5^

Dietary effects are tightly coupled to interactions with the gut microbiome, which begins to establish at birth (and possibly *in utero*) and profoundly influences metabolic, immune, endocrine, and neurological development.^6^ ^,^ ^7^ Microbial metabolites such as short-chain fatty acids (SCFAs) can cross the placenta and regulate embryonic organ differentiation via G-protein-coupled receptors.^8^ Early-life microbiome architecture is shaped by pregnancy, parturition, and infancy and can have lasting consequences.^9^ Western-style dietary patterns rich in fats and simple sugars exacerbate age-associated disease risk and adverse transgenerational outcomes, in part by promoting gut dysbiosis.^10^ For instance, maternal BMI has been linked to adverse shifts in neonatal communities, including higher proportions of *Clostridium*, *Staphylococcus*, and *Bacteroides.* ^11^ Because infant microbial taxa often persist into adulthood, diets rich in fiber, PUFAs, and polyphenols are critical for gut homeostasis and healthy aging.^12^

Among macronutrients, dietary fats exert a pronounced influence on microbiota composition. Lard-derived lipids have been associated with enrichment of *Bacteroides* and *Bilophila*, Toll-like receptor–driven inflammation, and impaired insulin sensitivity.^13^ In contrast, fish-derived lipids favor beneficial taxa such as *Bifidobacterium*, *Lactobacillus*, and *Akkermansia* and mitigate inflammatory and metabolic disturbances. Recent studies have highlighted the role of polyunsaturated fatty acids (PUFAs), especially omega-3 (n3) fatty acids, in shaping developmental trajectories of the brain, immune, cardiovascular, and metabolic health.^14^ ^,^ ^15^ This underscores the importance of ensuring adequate n3 intake, which in the United States often falls below recommended levels, particularly among socioeconomically disadvantaged groups.^16^ Prenatal and postnatal n3 supplementation confers benefits through multiple mechanisms, with gut microbiome modulation emerging as a key factor.^17^ ^,^ ^18^ Some of the studied mechanisms of n3-fatty acids in host physiology include reprogramming fetal immunity to reduce allergic responses,^19^ attenuating placental inflammation in maternal endothelial cells,^20^ ameliorating lipopolysaccharide (LPS)-induced microglial neuroinflammation in offspring,^21^ enhancing brain volumes in subregions of the corpus callosum and frontal cortex,^22^ and increasing butyrate-producing bacteria while decreasing pathogenic taxa.^23^ Collectively, these findings support the potential of maternal n3 intake to improve transgenerational neurocognitive and metabolic outcomes across the life course.

Despite these advances, limited knowledge exists on how maternal PUFA sources (n3 vs. n6) reshape offspring gut microbiota and how such restructuring influences long-term metabolic and neurodevelopmental trajectories. Herein, we hypothesize that an n3-enriched maternal diet would mitigate adverse Western-style diet (WD)-induced stressors post-weaning, promoting healthier gut–brain development and conferring transgenerational advantages. To test this hypothesis, dams were fed isocaloric WD differing in fat sources, and offspring were exposed to WD post-weaning through adulthood. We assess maternal dietary influences on offspring metabolism, neurodevelopment, and the gut microbiome, alongside fecal-serum-brain metabolomes and brain-lipidomes, followed by their immunomodulatory role in the gut–brain–immune axis using western blots and human microglial cells.

## Materials and methods

### Animal models and ethics

**Figure 1.**
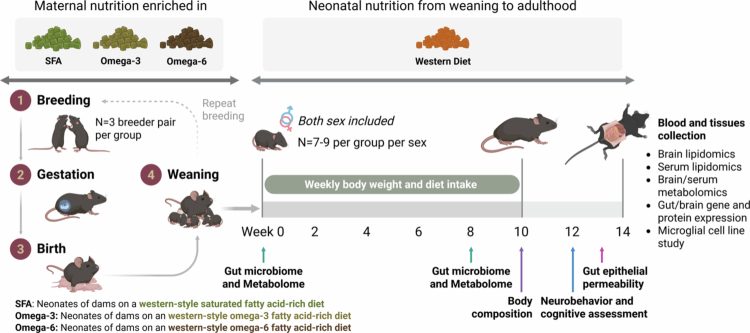
Overview of the experimental design.

C57BL/6J mice breeders (*n* = 24; F/M: 12/12) were procured at approximately 8 weeks of age from The Jackson Laboratory, ME. Following a 2-week acclimation period on standard rodent chow, the mice were randomized to establish a total of 12 independent breeding pairs assigned to four groups (3 breeding pairs per group) and were maintained on an irradiated isocaloric and iso-nitrogenous WD blended with either: 20% w/w anhydrous milk fat (saturated fatty acid diet, SFA); 20% w/w corn oil (omega-6 fatty acid diet, n6); or 19% w/w olive oil plus 1% w/w fish oil (omega-3 fatty acid diet, n3); or a Mediterranean-style diet (MD) (refer to Supplementary Table S1 for composition). The breeding pairs underwent regular health checks (biweekly) to monitor for pregnancy and litter presence. All breeding pairs across dietary groups were initiated simultaneously to ensure comparable timing of dietary exposure prior to conception. Upon confirmation of pregnancy, indicated by observable weight gain in the females,^24^ males were transferred to individual cages while the females remained undisturbed in their original cages. Both male and female mice continued their respective diets throughout breeding. Following parturition, the pups were weaned on post-natal day 21 and housed separately by sex (*n* = 3‒4/cage). The breeding pairs were reunited to produce second and third litters (triplicate litters), subjected to the same diet interventions as the first litter until sufficient pups were obtained (*n* = 7-9/group/sex). Furthermore, during subsequent breeding cycles, dams were immediately re-mated to minimize variation in dietary exposure intervals between successive pregnancies. This finding was confirmed as no significant differences were observed among the groups for exposure duration from breeding to delivery and the total dietary exposure duration from breeding until weaning (Supplementary Table S3). The offspring were exposed to the experimental diets *in utero* until birth and through breast milk for three additional weeks. Post-weaning, pups continued the WD until 10 weeks of age. Offspring from all three breeding cycles were proportionally and equally allocated across the experimental groups, with litter sizes ranging from 5 to 7 pups among the dietary groups (Supplementary Table S4). The overall experimental design used to understand the influence of maternal dietary fat sources (SFA, n3, and n6) on offspring health is summarized in Figure 1.

Bodyweight and diet intake were monitored weekly until 10 weeks. Fecal samples were collected at weaning (Wng) and 8 weeks for microbiome analysis. Between 8–12 weeks of age, offspring underwent neurobehavioral tests with 2–3 d of rest between sessions. Gut permeability was assessed at the endpoint. The mice were euthanized under isoflurane anesthesia via cervical dislocation (IACUC, FSU), and the blood and intestinal tissues were stored at –80 °C. The serum was used for lipid profiling, the ileum and hippocampus for gene/protein expression, the feces/serum/brain for metabolomics, and the brain for lipidomics. All procedures were approved by the Institutional Animal Care and Use Committee at Florida State University (Protocol #PROTO202100008).

### Bodyweight, food intake, and body composition

Weekly assessments were conducted to measure body weight and diet consumption. Additionally, body composition, including lean and fat mass, was evaluated at the end of the study using the EchoMRI-130 Body Composition Analyzer (EchoMRI, Houston, TX, USA).

### Microbiome analyses

The gut microbiome was assessed using the methodologies described in the previous studies.^25^ ^,^ ^26^ Briefly, genomic DNA from the fecal specimens was first extracted by using the QIAmp PowerFecal Pro DNA Kit. To target the hypervariable V4 region of the bacterial 16S rRNA gene, the universal barcoded primers 515F and 806R were employed. Library preparation involved paired-end sequencing on an Illumina platform, adhering to the Earth Microbiome Project benchmark protocol. After amplification, the resulting amplicons were purified with AMPure® (Agencourt) magnetic beads, and the purified product was quantified using a Qubit-4 fluorimeter (Invitrogen). Equal molar concentrations of the finalized amplicon library were then pooled and subjected to paired-end (2 × 300 bp) sequencing on an Illumina MiSeq system (Miseq Reagent Kit v3; Illumina Inc., San Diego, CA, USA) at the Translational Science Laboratory, College of Medicine, FSU. Bioinformatics processing of the microbiome data was carried out using QIIME2 (version 2-2023.5), encompassing demultiplexing and quality filtering (via the q2-demux plugin), followed by read trimming and denoising using DADA2.^27^ All identified amplicon sequence variants (ASVs) were aligned with MAFFT, and taxonomic classification was performed through a scikit-learn-based classifier trained on either the 99% SILVA 138 database.

### Metabolomic analyses

Fecal, serum, and brain samples underwent extraction using water, following a previously established protocol,^28^ with minor adjustments as in our earlier studies.^26^ ^,^ ^29-31^ To ensure a representative and uniform tissue sample from the brain, which is a highly heterogeneous organ, the whole brain was thoroughly homogenized, and aliquots of the homogenate were subsequently used for metabolomic analyses. Briefly, each sample was vortexed for 5 min in deionized water, and the resulting extracts were combined with phosphate buffer (pH 7.4) in deuterium oxide (D₂O). This process yielded a solution containing 10% D₂O, 0.1 M phosphate, and 0.1 mM trimethylsilyl propionate (TSP). After centrifugation, the samples were placed into 5 mm NMR tubes and analyzed using a Bruker Ascend 400 MHz high-resolution NMR (Bruker BioSpin, Germany). A 1D NOESY experiment with water suppression (64 scans) was performed on each sample. Subsequently, all NMR spectra were phased and calibrated to TSP in TopSpin 4.06 (Bruker BioSpin), followed by data processing in Amix 4.0 (Bruker BioSpin). The spectra were bucketed using an automated method designed to reduce peak overlap, and metabolite identification was conducted in Chenomx 8.6 (Chenomx Inc.). Prior to further analysis, total intensity normalization was applied to determine the concentrations of the individual metabolites.

### Lipidomics analyses

The brain tissues were homogenized in 1 mL of ethanol containing 2 g/L butylated hydroxytoluene (BHT) using a BeadMill 4 (Fisherbrand, USA) and then centrifuged at 14,000  rpm for 10 min at 4 °C. A 100 µL portion of the supernatant was transferred to a glass vial. Free fatty acid levels were determined via an Agilent Technologies 1290 Infinity II Series UHPLC coupled to a 9465C Triple Quadrupole mass spectrometer (Agilent, Santa Clara, CA, USA) operating in electrospray ionization (ESI) negative MRM mode at the Metabolomics Core, University of Illinois (IL). Chromatographic separation employed an Acquity BEH C18 column (100 × 2.1 mm, 1.7 µm; Waters, USA) with a two-solvent gradient. Mobile phase A consisted of 5 mM ammonium acetate in a 7:3 (v/v) mixture of water:methanol with 0.05% acetic acid, while mobile phase B contained 5 mM ammonium acetate in a 4:6 (v/v) mixture of IPA:methanol with 0.05% acetic acid. The flow rate was maintained at 0.3 mL/min, and the peaks were integrated and quantified using MassHunter 12.1 software (Agilent, USA).

### Gut permeability

Gut permeability was assessed using the oral administration, followed by serum detection of fluorescein isothiocyanate (FITC)-dextran as per our previously described methods.^32^ ^,^ ^33^ Briefly, the mice underwent a 4-hour fasting period before receiving an oral gavage of FITC-dextran solution at a dosage of 60 mg/100 g body weight. Approximately 50  μL of blood from the tail tip was collected into a heparinized capillary tube 2 h after FITC administration. The concentration of FITC-dextran in the serum was assessed using fluorescence spectroscopy at 530 nm with excitation at 485 nm using a plate reader.

### Neurobehavioral assessments

#### Open-field test

General locomotor activity was evaluated in a dedicated procedure room within the vivarium, as previously described.^34^ Mice were gently introduced into a sanitized 40 × 40 cm box, and their movements were recorded by video for 5 min. Ethovision XT software (Noldus) was used to measure behavioral parameters, including mobility, activity, total distance traveled, and the time spent (%) in the central area.

#### T-maze spontaneous test

Spatial working memory was evaluated using a T-maze design based on an established method.^35^ Briefly, the mice were placed at the far end of the start arm, oriented toward the south wall, and allowed to move freely. The choice of the left or right arm in the goal area was recorded over seven trials, with the maze cleaned using 70% ethanol after each trial. Working memory was quantified through the percent alternation score, computed as: Total correct alterations/6 × 100.

#### Location memory test

The experiment adhered to the previously outlined protocol,^36^ with several adjustments. Briefly, four distinct objects were placed at equal distances within an open-field arena. The mice were gently positioned at one end of the arena, facing a wall, and allowed to explore for 5 min. After 24 h, two adjacent objects were swapped, and spatial memory retention was assessed over a 5-minute video session. Ethovision XT software recorded the time each mouse spent exploring objects when the mouse’s nose was within 2 cm. Memory retention was calculated using the discrimination index: (time with relocated objects)—(time with familiar objects)/(total exploration time).

### mRNA expression assays

Total RNA from frozen tissues was extracted using RNeasy kit as per manufacturer's instructions. The mRNA concentration was measured using NanoDrop One (Thermo Scientific). The concentration of RNA from all the tissues was normalized either to 50 or 100 ng/µL for further reverse transcription using the high-capacity cDNA reverse transcription kit. The cDNA obtained was diluted 8–10-fold before performing relative mRNA expression using qPCR (QuantStudio3, Applied Biosystems). The Apex 2X Green Master Mix (Genesee Scientific, NC) was used for a 10-step qPCR reaction with the following conditions: an initial denaturation step at 95 °C for 15 min, followed by 40 cycles of 15-s denaturation step at 95 °C and a 60-s annealing step at 60 °C. A melt curve analysis was performed for each primer pair to ensure that a single product was efficiently amplified. The details of the primer sequences used are listed in Supplementary Table S2. The 18S gene was employed as an internal housekeeping control for normalization, and the results were expressed via the ddCt method.

### Serum lipids

Serum lipids (total cholesterol, high-density lipoprotein (HDL), low-density lipoprotein (LDL), very low LDL (VLDL), and triglyceride (TG)) were measured using the Piccolo Xpress Chemistry Analyzer (Abaxis, USA) by utilizing a Piccolo Lipid Panel Plus Reagent Disc as per the manufacturer’s protocols.

### Western blots

Hippocampal and ileal tissues were homogenized (2000 rpm, Heidolph RZR2021) in Buffer A (50 mM HEPES, pH 7.4; 150 mM NaCl; 1 mM EGTA; 0.1 mM MgCl₂) with 1% Triton X-100, phosphatase inhibitors (2 mM sodium orthovanadate, 10 mM sodium pyrophosphate, 10 mM sodium fluoride), and a protease inhibitor cocktail (Sigma, P340-5 ML). The lysates were incubated on ice for 20 min with vortexing, centrifuged, and the supernatants were collected. The protein concentration was determined at 595 nm using a Bradford assay (Eppendorf BioPhotometer plus). Equal amounts (50 µg) were resolved on 4%–20% Criterion gels (BioRad) and transferred to PVDF membranes (Thermo Scientific) using a semi-dry transfer system. The membranes were blocked with 1.5% nonfat dry milk in TBS, then incubated overnight at 4 °C with primary antibodies against IL1β, IL6, TNFα, MCP1, GFAP, and GAPDH (Invitrogen; 1:1000 dilution in 3% BSA with 0.1% sodium azide). After TBS-T washes, HRP-conjugated secondary antibodies were applied for 45  min, and the bands were visualized using Pierce ECL substrate on an Azure 300 chemiluminescent system.

### Tissue histology

Ileum tissues were fixed in 10% buffered formalin (48 h), transferred to 70% ethanol, and processed for paraffin embedding using an automated tissue processor (FSU College of Medicine). The tissues were dehydrated (70%–100% ethanol), cleared (xylene), infiltrated with paraffin, and embedded (Tissue-Tek TEC, Sakura, CA, USA). Sections (5 µm) were cut on a microtome (HM 340E, Epredia, CA), mounted on glass slides, and dried at 35 °C overnight. For H&E staining, the slides were deparaffinized (xylene, ethanol), rinsed with DI water, stained with Mayer’s hematoxylin (5 min), differentiated in acid alcohol, washed, and counterstained with eosin (5 min). After dehydration and clearing, the slides were mounted with Permount medium (Electron Microscopy Sciences, PA), coverslipped, and imaged at 10× magnification on a Leica DMI4000B microscope using Axiovision Rel 4.5 software.

### Multiplex ELISA analysis

Serum MCP-1 and S100A9 were quantified using the Mouse Premixed Multi-Analyte Luminex® Discovery Assay (Cat # LXSAMSM, Lot # L151578, R&D Systems, MN) following the manufacturer’s instructions. Briefly, 50 µL of serum was incubated with antibody-coated microparticles (2 h, RT, 800 rpm), washed, and then incubated with 50 µL of biotinylated antibodies (1 h, RT) followed by Streptavidin-PE (30 min, RT). After the final washes, the microparticles were resuspended in 100 µL of wash buffer, and fluorescence was measured within 90  min using the Luminex MAGPIX CCD Imager (Luminex Corp, TX, USA).

### HMC3 cell culture assays

The HMC3 cell line (ATCC® CRL-3304™) was cultured in EMEM (Corning) with 10% FBS (VWR) and 1% penicillin–streptomycin–L-glutamine (Corning) at 37 °C and 5% CO₂. The cells were passaged at ~80% confluency, trypsinized with TrypLE Express (Gibco), and centrifuged (800 × g, 5  min). At passage 7, 2.16 × 10⁴ cells/well were seeded in 12-well plates, incubated for 24 h, and then starved in serum-free medium for 24 h. The cells were treated with 10% mouse serum from the diet groups or 10% FBS (control) for 24 h. RNA was extracted, cDNA was synthesized, and qPCR was performed for BDNF-signaling and microglial inflammation markers, with GAPDH used as the reference gene (primer sequences in Supplementary Table S2).

### Statistical and bioinformatic analyses

Data are expressed as the mean ± SEM. Statistical analyses were conducted in SPSS (v29.0.1.0), with significance at *p* < 0.05. Depending on the distribution, one-way ANOVA or the Kruskal‒Wallis test with Dunn’s post-hoc test and FDR correction was applied. GraphPad Prism (v10) was used for visualization. Neurobehavioral and lipid data were analyzed by ANCOVA (adjusted for sex) with LSD post-hoc tests. Effect sizes (Cohen’s d, 95% CI) were calculated via Student’s t-test. Microbiome analyses were performed in R or Python. Alpha diversity was estimated with Chao1 (richness) and Shannon (richness/evenness) indices; beta diversity was estimated with Bray‒Curtis dissimilarity and PCoA. Group differences were tested using Kruskal-Wallis,^37^ and PERMANOVA (999 permutations).^38^ Supervised classification was performed using the q2-sample-classifier (Random Forest, 5000 trees, nested stratified 5-fold cross-validation).^39^ Differential taxa and functions were identified via LEfSe.^40^ Correlations were assessed with Spearman’s rank, and microbial networks were visualized with CoNet in Cytoscape. Metagenomic functional potential was predicted using PICRUSt2 (Phylogenetic Investigation of Communities by Reconstruction of Unobserved States 2).^41^ Amplicon sequences were used as input to infer the functional gene content of microbial community members based on phylogenetic placement. The predicted gene families were annotated against Kyoto Encyclopedia of Genes and Genomes (KEGG) orthologs and subsequently collapsed into KEGG pathways to generate functional pathway profiles. Differential abundance analysis of the predicted functional pathways was conducted using MaAsLin3 (Multivariable Association with Linear Models 3).^42^ A multivariable linear modeling framework was applied including group, sex, and age as fixed effects. Associations between diet groups and functional pathways were estimated using models adjusted for sex and cage effects. Default normalization and transformation procedures implemented in MaAsLin3 were applied. Multiple hypothesis testing correction was performed using the Benjamini‒Hochberg false discovery rate (FDR), and adjusted q-values were reported. SourceTracker2 was employed to estimate the proportion of the gut microbiome transmitted from breeders to their offspring and maintained up to 8 weeks of age.^43^ An amplicon sequence variant (ASVs) table was used as input, and the model was applied to estimate the relative contributions of the breeder gut microbiota (defined as source communities) and unknown sources to the infant gut microbiome (defined as sink communities). For this analysis, each infant was paired exclusively with its corresponding breeder, ensuring one-to-one matching between source and sink samples.

### Role of funders

The funders of the study had no role in the study design, data collection, statistical analysis, results interpretation, or writing of the report.

## Results

### Maternal n3-rich diet confers neonatal physiological and metabolic benefits

**Figure 2.**
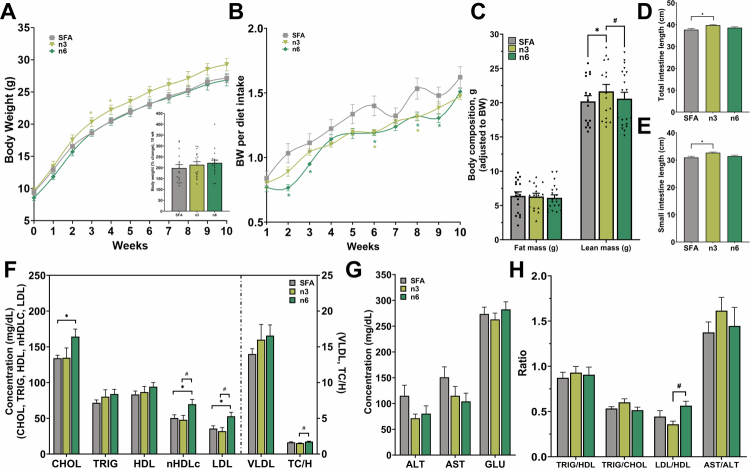
Maternal diet differing in fatty acid sources differently shapes neonatal physiological and metabolic development. (A) Weekly bodyweight (g). (B) Weekly bodyweight per diet intake. (C) Body composition adjusted to bodyweight for fat mass (g) and lean mass (g). (D) Total intestinal length (cm). (E) Small intestinal length (cm). (F) Circulating lipoproteins concentration (mg/dL). (G) Hepatic markers and glucose concentration (mg/dL). (H) Lipid ratios. CHOL: Cholesterol; TRIG: Triglycerides; HDL: High-density lipoprotein cholesterol; LDL: Low-density lipoprotein cholesterol; VLDL: Very-low-density lipoprotein cholesterol; TC/H: Total cholesterol to HDL ratio; ALT: Alanine aminotransferase; AST: Aspartate aminotransferase; GLU: Glucose. *p < 0.05 between SFA and PUFA groups, ^#^ *p* < 0.05 between n3 and n6 groups, assessed using ANCOVA after adjusting for sex with LSD post-hoc analysis. The data are presented as the mean ± SEM; *n* = 16–20 mice/group, except for those in panels F–H, where cage-wise pooled serum samples were used (*n* = 6–7 pooled samples/group).

The overall impacts of SFA- and PUFA-enriched maternal diets on the physiological and metabolic outcomes of offspring are summarized in Figure 2. The offspring of the n3 dams consistently maintained a higher bodyweight throughout the 10 weeks of the WD feeding regimen, with significantly higher (*p* < 0.05) weight observed at weeks 3 and 4, compared to the SFA group (Figure 2A). However, there was no significant difference in the bodyweight (% change) of offspring among the different groups over the 10-week feeding period. Besides, both PUFA groups had lower bodyweights when adjusted for diet intake relative to the SFA group (Figure 2B). Notably, the n3 group demonstrated significantly higher (*p* < 0.05) lean mass compared to the SFA and n3 groups, with no significant differences in fat mass (Figure 2C). In terms of tissue measurements, the n3 exhibited significantly longer (*p* < 0.05) total intestinal lengths, including the small intestine, compared to the SFA group (Figure 2D–E). Lipid metabolism was also differently impacted by the different diet groups. The n6 group induced hyperlipidemia, with significantly increasing (*p* < 0.05) CHOL relative to the SFA group and significantly increasing (*p* < 0.05) nHDLc and LDL levels relative to both SFA and n3 groups. The n6 group also significantly enhanced (*p* < 0.05) the TC/H ratio relative to the n3 group (Figure 2F). There was no prominent effect of the groups on markers of hepatic health; however, the levels of ALT and AST were suppressed in both PUFA groups compared to the SFA group (Figure 2G). Moreover, the ratio of different lipid profiles largely remained insignificant, except for the LDL/HDL ratio, which was increased significantly (*p* < 0.05) in the n6 group relative to the n3 group (Figure 2H).

### Maternal n3-intake shapes neonatal microbiome signatures, which persist till adulthood

**Figure 3.**
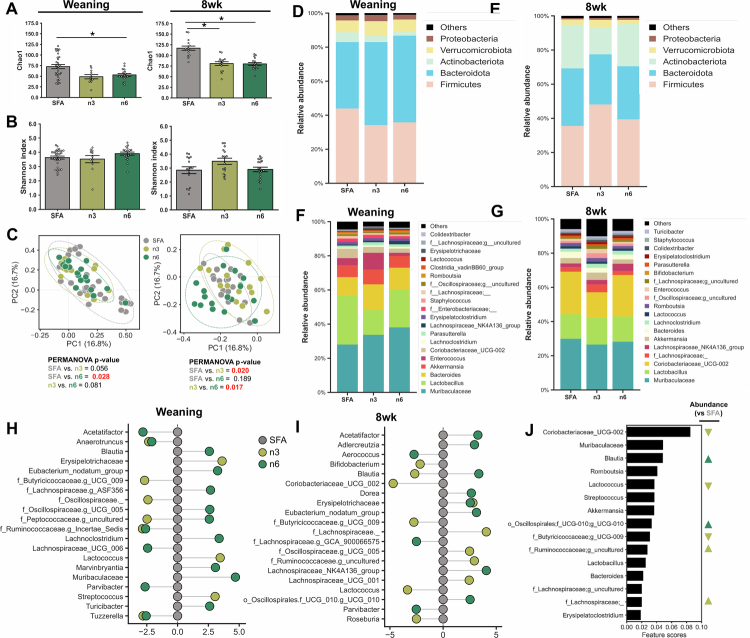
Maternal diet differing in fatty acid profiles differently fosters the neonatal gut microbiome. Alpha-diversity was assessed at weaning and 8-weeks using (A) the Chao1 index and (B) the Shannon index. (C) Beta-diversity was assessed at weaning and 8 weeks using Bray-Curtis PCoA analysis, with significance determined by PERMANOVA (*p* < 0.05). Phylum-level taxonomic relative abundance at (D) weaning and (E) 8 weeks. Top 20 genera-level taxonomic relative abundance at (F) weaning and (G) 8 weeks. Linear discriminant analysis effect size (LEfSe) with an LDA score ≥ 2.0 and *p* < 0.05 showing group-specific discriminatory taxa at (H) weaning and (I) 8 weeks. (J) Machine learning using the Random Forest classifier showing the top 15 features for group-specific prediction, with the directionality of relative abundance highlighted by group-specific-colored arrows, compared to the SFA group as per LEfSe analysis. *q < 0.05. The data are presented as mean ± SEM; *n* = 16–20 mice/group.

We examined how replacing SFA in a Western-style maternal diet with n3- or n6-rich PUFAs during the periconceptional, prenatal, and perinatal periods shapes the neonatal gut microbiome at weaning and whether these effects persist when offspring consume a WD into adulthood. The overall impacts of maternal diet differing in fat sources on the neonatal gut microbiome are summarized in Figure 3. The n3 and n6 groups exhibited significantly lower (*q* < 0.05) Chao1 *α*-diversity after 8 weeks of WD feeding compared to the SFA group, while only the latter group exhibited significantly lower *α*-diversity right after weaning (Figure 3A). There were no significant variations in the Shannon *α*-diversity in either of the treatment groups compared to the SFA group (Figure 3B). The *β*-diversity at weaning was significantly changed for the n6 group (*p* = 0.028), while it was nearly significant for the n3 group (*p* = 0.056) when compared to the SFA group. Notably, the n3 group exhibited significantly different *β*-diversity relative to the SFA (*p* = 0.020) and the n6 (*p* = 0.017) groups, while this distinction was non-significant between the n6 and SFA groups. (Figure 3C). At the phylum level, the treatment groups showed a lower proportion of Bacillota (formerly Firmicutes) and a higher proportion of Bacteroidota compared to the SFA group at weaning. However, this trend reversed after 8 weeks of WD feeding. Additionally, there was a bloom of the Actinobacteria phylum from weaning to 8 weeks across all groups (Figure 3D–E). At the genus level, the top 20 genera at the weaning and 8 weeks were selected for visualization (Figure 3F–G). The genera, namely, *Muribaculaceae*, *Lactobacillus*, and *Bacteroides*, comprised 60%–70% of the total abundance among the selected genera at weaning. At 8 weeks, the former two, along with *Coriobacteriaceae_UCG-002*, occupied a similar proportion.

To further identify group-specific discriminative taxa at weaning and 8 weeks, we performed LEfSe analysis (Figure 3H–I). Thirty-two genera were differentially abundant across groups at two time points, with 8 genera present at both time points. Among them, the group-specific key genera that maintained consistently higher abundance from weaning to 8 weeks included *Blautia* and *Eubacterium_nodatum_group* (n6 group), *Erysipelotrichaceae* (n3 group), and *f_Butyricicoccaceae;g_UCG009* and *Parvibacter* (SFA group). Notably, a higher abundance of *Erysipelotrichaceae* was also observed in the dams fed the n3-enriched diet post LEfSe analysis (Supplementary Figure S1F). Similarly, other n3-associated taxa, including *Lactococcus* and *Streptococcus*, were enriched in dams and were also elevated in offspring at weaning, although these differences were not maintained after 8 weeks. Moreover, source tracking analysis demonstrated that approximately 60%–80% of the ASVs were retained across dietary groups from weaning to the 8-week time point (Supplementary Figure S2). These findings collectively suggest potential vertical transmission and early-life persistence of specific maternal microbial features. A machine learning approach using the Random Forest model was applied to taxa at 8 weeks to identify the top 15 taxa with the highest feature importance scores explaining the differences among groups. Among them, *Coriobacteriaceae_UCG-002*, *Blautia, Lactococcus*, *f_Butyricicoccaceae;g_UCG_009*, f*_Ruminococcaceae;g_uncultured*, and *f_Lachnospiraceae;_* were also identified as discriminatory taxa by LEfSe analysis (Figure 3J).

Further, we also performed PICRUSt2-based functional interpretation of the microbiome, which revealed differential regulation of several pathways associated with energy, lipid, carbohydrate, amino acid, and xenobiotic metabolism between the SFA and n3 groups (Supplementary Figure S3). Specifically, the SFA group showed enrichment of pathways linked to energy harvesting, oxidative stress, and inflammation, including oxidative phosphorylation, steroid biosynthesis, lipoic acid metabolism, the phosphotransferase system, and *Staphylococcus aureus* infection pathways.^44-47^ In contrast, the n3 group exhibited enrichment of pathways related to D-arginine and D-ornithine metabolism, sphingolipid metabolism, flavonoid biosynthesis, and insulin signaling, which are associated with improved host metabolism and reduced chronic inflammation.^48-50^ Although PICRUSt2 provides only predictive functional inference rather than direct measurement of microbial activity, these findings offer supportive evidence for maternal diet-associated functional remodeling of the neonatal gut microbiome.

### Maternal diet modulates gut, blood, and brain metabolomic arrays

**Figure 4.**
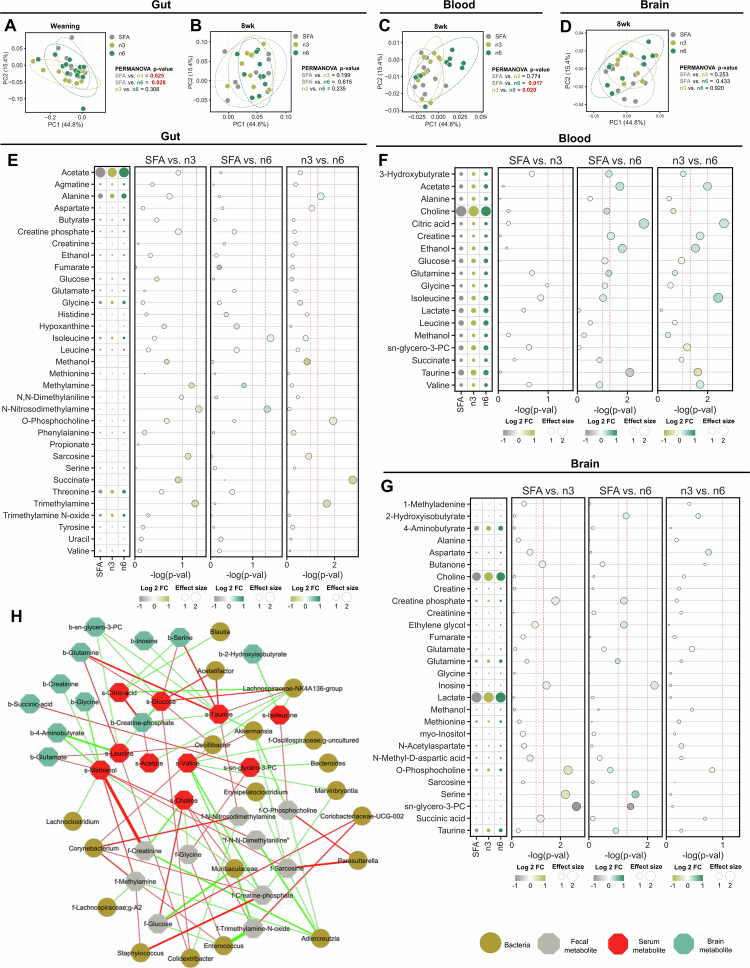
Maternal diet differing in fat sources differently influences neonatal fecal, serum, and brain metabolomic arrays. Beta-diversity was assessed using Bray–Curtis PCoA for the (A) fecal metabolome at weaning, (B) fecal metabolome at 8 weeks, (C) serum metabolome at 8 weeks, and (D) brain metabolome at 8 weeks. Statistical significance was determined by PERMANOVA (*p* < 0.05). Abundance and bubble plot at 8 weeks for the (E) fecal metabolome, (F) serum metabolome, and (G) brain metabolome. The size of each bubble represents Cohen’s d effect size, and the color intensity indicates the log2-fold change between the two groups. Significance (*p* < 0.05) was calculated using the Mann‒Whitney U test and plotted as a -log (*p*-value) for each metabolite. (H) Multi-omics correlation analysis identifies interconnections among microbiome-fecal-serum metabolome arrays and fecal-serum-brain metabolome arrays. Associations with an absolute Spearman’s coefficient > 0.4 and *p* < 0.05 are included for network visualization. The colored nodes denote different lineages, while edge colors indicate the association direction (green: positive; red: negative). Edge thickness reflects the strength of the association. The data are presented as mean ± SEM; *n* = 9–13 mice/group for panel A and *n* = 9–10 mice/group for panels B–H.

A total of 33, 18, and 28 metabolites were detected in the gut (feces), blood (serum), and brain samples after untargeted NMR metabolomics. The overall comparison of the fecal, serum, and brain metabolome profiles among the three groups is summarized in Figure 4. We first investigated the impact of maternal fat sources, rich in SFA and PUFA, on the offspring’s overall metabolome profile in fecal, serum, and brain samples using PCoA. The fecal metabolome profiles of the n3 (*p* = 0.025) and n6 (*p* = 0.028) groups were significantly different from the SFA group at weaning; however, no differences were observed after 8 weeks (Figure 4A, B). The serum metabolome profile of the n6 group was significantly different for the SFA (*p* = 0.017) and n3 (*p* = 0.020) groups, while no significance was noted between the n3 and SFA groups (Figure 4C). No significant differences for the brain metabolome profile were observed in any of the groups (Figure 4D).

Next, we examined how the relative abundance of individual metabolites differed among the groups after 8 weeks for each biological sample, using log2 fold change. The magnitude of these differences was evaluated using Cohen’s *d* effect size (Figure 4E‒G). Among the fecal metabolites, acetate, alanine, threonine, glycine, trimethylamine *N*-oxide (TMAO), and isoleucine were the most prominent, based on their high relative abundance (Figure 4E). *N*-Nitrosodimethylamine was significantly promoted (*p* < 0.05) in the n3 and n6 groups relative to the SFA group. The same metabolite was also significantly enriched in dams fed an n3-enriched diet (Supplementary Figure S1H). Trimethylamine (TMA), methylamine, and sarcosine exhibited a trend toward higher abundance in the n3 group against the SFA group (*p* < 0.10). Additionally, succinate and creatine phosphate were more abundant in the n3 group, and despite non-statistically significant differences, they demonstrated a large effect size (*d* > 0.8), indicating a potentially meaningful biological relevance despite the lack of significance. Isoleucine was significantly higher in the n6 group compared to the SFA group. Compared to the n6 group, the n3 group revealed a significantly higher proportion of succinate, TMA, and O-phosphocholine (OPC), but a lower proportion of alanine.

Among the serum metabolites, choline was the most abundant, followed by taurine, leucine, lactate, and sn-glycero-3-phosphocholine (snGPC) (Figure 4F). None of the metabolites exhibited significant variations between the n3 and SFA groups; however, isoleucine, which was higher in the SFA group, exhibited a larger effect (*d* > 0.8). The n6 group revealed significantly (*p* < 0.05) higher levels of citric acid, acetate, creatine, and ethanol but a lower level of taurine compared to both the SFA and n3 groups. Additionally, the n6 group revealed significantly (*p* < 0.05) and moderately (*p* < 0.10) higher proportion of isoleucine compared to the n3 and SFA groups, respectively. Besides, the levels of 3-hydroxybutyrate, glutamate, and glycine showed a higher trend, while choline and glucose exhibited a lower trend (*p* < 0.10) than that of the SFA group. Compared to the n3 group, the n6 group demonstrated a significantly higher prevalence of valine (*p* < 0.05) and a moderately higher prevalence of 3-hydroxybutyrate (*p* < 0.10), while showing moderately lower levels of glucose and snGPC (*p* < 0.10).

Among the brain metabolites, lactate and choline were the most abundant, followed by 4-aminobutyrate, taurine, and glutamine (Figure 4G). Creatine phosphate, inosine, and snGPC were presented in significantly upregulated proportions (*p* < 0.05) in the SFA group compared to the other two groups. Serine exhibited a significantly higher (*p* < 0.05) abundance in both PUFA groups; however, OPC was markedly more abundant (*p* < 0.05) in the n3 group, compared to the SFA group. Butanone and succinic acid were moderately higher (*p* < 0.10) with a larger effect size (*d* > 0.8) in the SFA group relative to the n3 group. The n6 group exhibited moderately higher (*p* < 0.10) abundance of 2-hydroxyisobutyrate and ethylene glycol than the SFA group.

Metabolomic analyses of dams further supported early-life programming effects (Supplementary Figure S1J, K). Serum levels of citric acid, creatine, and succinate tended to be higher in offspring born to n6-fed dams, and similar metabolic patterns persisted into adulthood after 8 weeks. Likewise, brain metabolite analysis in weaned pups demonstrated higher levels of sn-GPC, inosine, creatine, butanone, and succinic acid in the SFA group, with similar trends persisting later in life. Although serum lipid profiling in dams did not reveal statistically significant differences among groups (Supplementary Figure S1L–N), the microbiome and metabolomic findings together suggest that maternal dietary fatty acid composition influences early microbial and metabolic programming in offspring.

### Maternal diet shapes multi-omics coregulation arrays in neonatal gut, blood, and brain niches

Correlation network analyses were performed to assess associations between microbiome signatures and fecal–serum metabolomic arrays, as well as their subsequent impact on brain metabolites (Figure 4H). For instance, fecal glucose and TMAO showed strong positive associations with *Enterococcus* and *Muribaculaceae*, while *Coriobacteriaceae-UCG-002* exhibited negative associations with these metabolites. Both glucose and TMAO were negatively associated with serum choline. Serum choline levels were positively correlated with fecal creatine and glycine. Fecal glycine and *Bacteroides* were negatively and positively linked to serum snGPC levels, respectively, which in turn were negatively associated with brain succinic acid. Serum leucine levels correlated with multiple microbiome signatures (negatively with *Colidextribacter* and *Oscillibacter*) and brain metabolites, including positive associations with 4-aminobutyrate, sn-GPC, and creatine phosphate, and negative associations with glutamate and glutamine. *Akkermansia* positively interacted with fecal OPC and sarcosine, while showing a negative association with valine and a positive association with taurine in serum. Serum taurine was negatively associated with brain serine and glutamine, but positively associated with brain snGPC and inosine. *Lachnospiraceae-NK4A136* influenced various serum metabolites, showing positive co-occurrence with isoleucine, valine, acetate, and citric acid, which in turn were associated with different brain metabolites, including 2-hydroxyisobutyrate, creatine phosphate, and serine. Collectively, these associations highlight the profound role of the microbiome in shaping both local and systemic metabolite pools.

### Maternal n3-rich diet improves neonatal gut epithelial and inflammatory features

**Figure 5.**
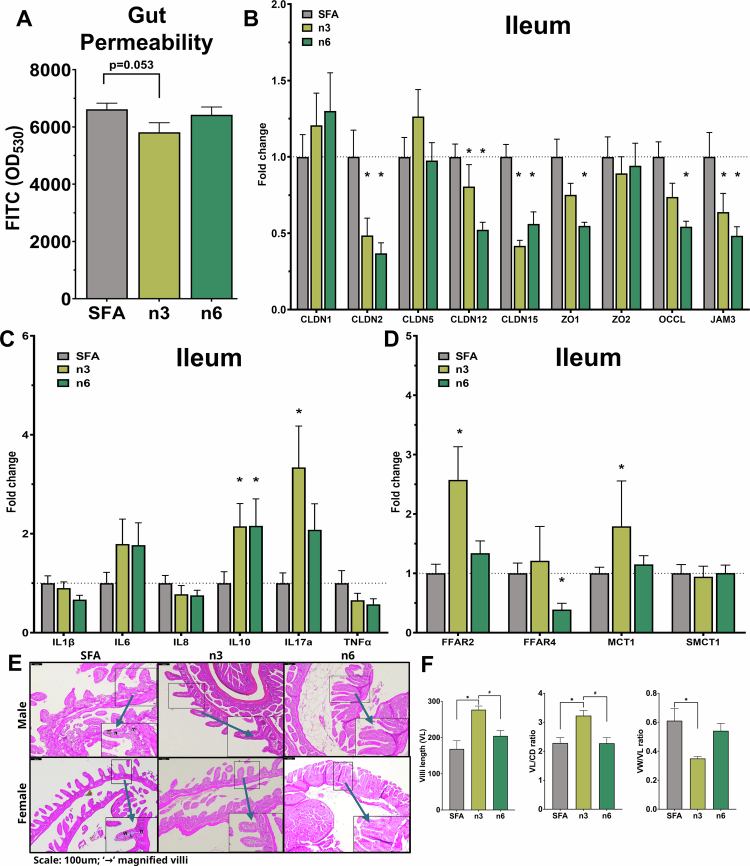
Maternal diet differing in fat sources differently shapes neonatal intestinal health. (A) Gut permeability using FITC-dextran assay (OD_530nm_). (B) mRNA expression of tight-junction proteins in the ileum. (C) mRNA expression of inflammatory markers in the ileum. (D) mRNA expression of fatty acid receptors and transporters in the ileum. (E) H&E-stained histological sections of ileum tissue. Scale bar: 100 µm. The arrows indicate representative magnified villi for each group, with measurements shown for width (W), length (L), and diameter (D). (F) Assessment of intestinal morphology for villi length, villi length-to-crypt depth ratio (VL/CD), and villi width-to-villi length ratio (VW/VL). *p < 0.05 between SFA and PUFA groups, ^#^ *p* < 0.05 between n3 and n6 groups, assessed using Kruskal-Wallis test with Dunn’s post-hoc analysis (non-parametric data) and One-way ANOVA with Tukey’s post-hoc analysis (parametric data). The data are presented as mean ± SEM; *n* = 14–19 mice/group for panel A, *n* = 14–17 mice/group for panels B–D, and *n* = 12 mice/group for panel F.

The alterations in gut permeability, along with the gene expression of tight-junction proteins (TJPs), inflammatory markers, and fatty acid receptors in the ileum, as well as villi morphological alterations, are summarized in Figure 5. Gut permeability, assessed by the intestinal transfer of 4 kDa-FITC-dextran from the lumen to the blood, exhibited a near-significant reduction (*p* = 0.053) for the n3 group compared to the SFA group (Figure 5A). Further investigation of changes in the gene expression of different tight junction proteins in the ileum revealed significant downregulation (*p* < 0.05) of pore-forming claudins (CLDN-2, 12, and 15) in both the PUFA groups compared to the SFA group (Figure 5B). There were no marked changes in the expression of the barrier-forming claudins (CLDN-1 and CLDN-5) in the ileum; however, a positive increasing trend was observed for both claudins in the n3 group. The gene expression of ZO1, a scaffolding protein, was significantly lower (*p* < 0.05) in the n6 group compared to the SFA group. Other barrier-forming proteins, OCCL and JAM3, were significantly downregulated (*p* < 0.05) in the n6 group compared to the SFA group, with a significant reduction (*p* < 0.05) of JAM3 also observed in the n3 group.

Among the inflammatory markers, the IL10 and IL17 genes were markedly upregulated (*p* < 0.05) by the n3 group compared to the SFA group in the ileum (Figure 5C). IL10 was also significantly increased (*p* < 0.05) for the n6 group, along with non-significant but observable upregulation of IL17 compared to the SFA group. We also investigated the expression of key free fatty acid receptors (FFAR2/4) and transporters (MCT1 and SMCT1) within the ileum (Figure 5D). Interestingly, the gene expression of FFAR2 and MCT1 was significantly upregulated (*p* < 0.05) by the n3 group than that of the SFA group. Besides, the expression of FFAR4 was downregulated by the n6 group compared to the SFA group. The morphological alterations in the ileum were assessed using H&E staining (Figure 5E, F). Villi length (VL) and the villi length-to-crypt depth (VL/CD) ratio were significantly increased (*p* < 0.05) in the n3 group compared to both the SFA and n6 groups. In contrast, the VW/VL ratio was significantly decreased (*p* < 0.05) in the n3 group compared to both the SFA and n6 groups.

### Maternal n3-intake shapes neonatal neurodevelopment

**Figure 6.**
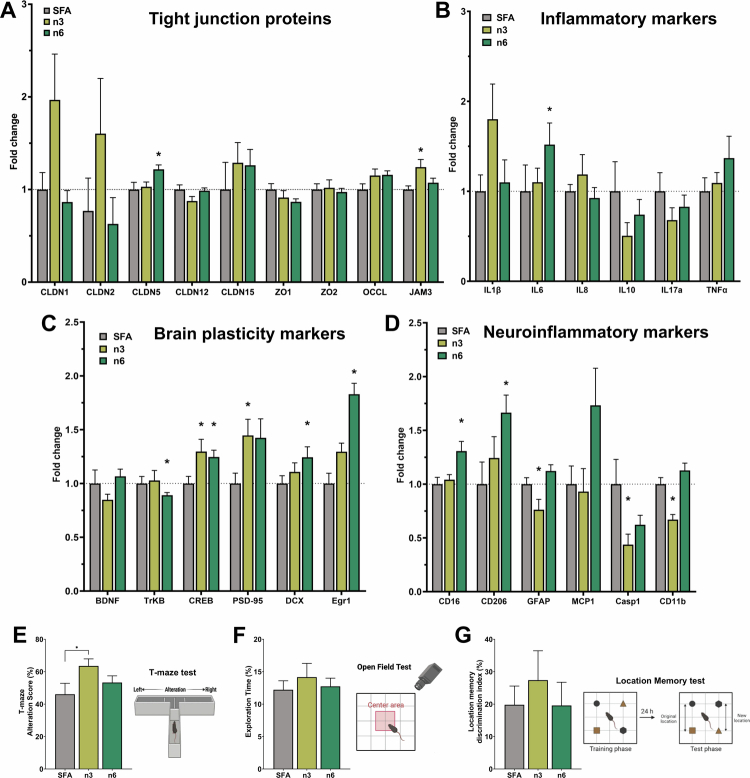
Maternal diet differing in fat sources differently shapes neonatal brain and cognitive development. mRNA expression analysis of (A) Tight-junction proteins in the hippocampus, (B) inflammatory markers in the hippocampus, (C) synaptic plasticity and neurodevelopmental markers in the hippocampus, and (D) neuroinflammatory markers in the hippocampus. (E) T-maze alteration score (%). (F) Exploration time (%). (G) T-maze latency (s). (I) Location memory discrimination index (%). Significance for mRNA expression analysis was calculated using the Kruskal–Wallis test with Dunn’s post-hoc analysis, whereas One-way ANOVA with Tukey’s post-hoc correction was used for neurobehavioral tests. *p < 0.05 between SFA and PUFA groups. The data are presented as mean ± SEM; *n* = 10–14 mice/group for panels A–D and *n* = 16–20 mice/group for panels E–G.

To evaluate the effects of periconceptional-perinatal exposures of maternal diets on the neurocognitive and neurodevelopmental features of offspring, we studied the gene expression profiles of TJPs, inflammatory markers, neurodevelopmental, and neuroinflammatory markers in the hippocampus, as well as evaluated the exploratory behavior, spatial learning, and memory functions using different neurobehavioral tests, which are summarized in Figure 6. In the hippocampus, CLDN5 and IL6 were significantly upregulated by the n6 group, while JAM3 was significantly upregulated by the n3 group compared to the SFA group (Figure 6A-B). Furthermore, we investigated how the gene expression of brain synaptic plasticity and neuroinflammatory markers changes in response to different maternal diet groups (Figure 6C, D). In the hippocampus, CREB, a transcriptional factor associated with BDNF neuronal signaling, was significantly upregulated (*p* < 0.05) by both PUFA groups compared to the SFA group (Figure 6C). Besides, other synaptic genes like PSD95 were significantly upregulated (*p* < 0.05) by the n3 group, and DCX and Egr1 were significantly upregulated (*p* < 0.05) by the n6 group, compared to the SFA group. In terms of neuroinflammation, markers of microglial inflammation (CD16 and CD206), were significantly upregulated (*p* < 0.05) by the n6 groups, whereas GFAP (an astrocyte activation marker) together with other inflammatory markers, such as Casp1 and CD11b, were significantly downregulated (*p* < 0.05) in the n3 group compared to SFA group (Figure 6D). MCP-1 (a chronic inflammation marker) was also visibly upregulated in the n6 group compared to other groups; however, statistical significance was not achieved. The neurobehavioral tests revealed phenotypic changes in cognition. The T-maze alternation score was notably increased (*p* < 0.05) in the n3 group compared to the SFA group (Figure 6E). Although non-significant, the open field exploration time and location memory discrimination index were relatively higher for the n3 group compared to the SFA group, indicating the potential positive effects of the n3 group in enhancing spatial learning and working memory recognition (Figure 6F‒G). However, upon stratification by litter, we observed that such effects were not consistently significant across all litters (Supplementary Figure S4). Specifically, the T-maze alternation score generally followed the overall trend observed in the pooled analysis, with statistically significant differences between the SFA and n3 groups becoming evident only in litter-3. In contrast, no significant differences were observed for the open field exploration time among any of the individual litters. Similarly, the location memory discrimination index showed a trend consistent with that of the pooled dataset only in litter-2 between the SFA and n3 groups (*p* < 0.10), without reaching statistical significance across all litters. Thus, future studies involving larger sample sizes, greater litter representation, and longitudinal behavioral assessments will be necessary to determine the robustness and reproducibility of these neurobehavioral effects.

### Maternal n3-rich diet modulates neonatal neurodevelopment via gut–brain–immune axis mechanisms

**Figure 7.**
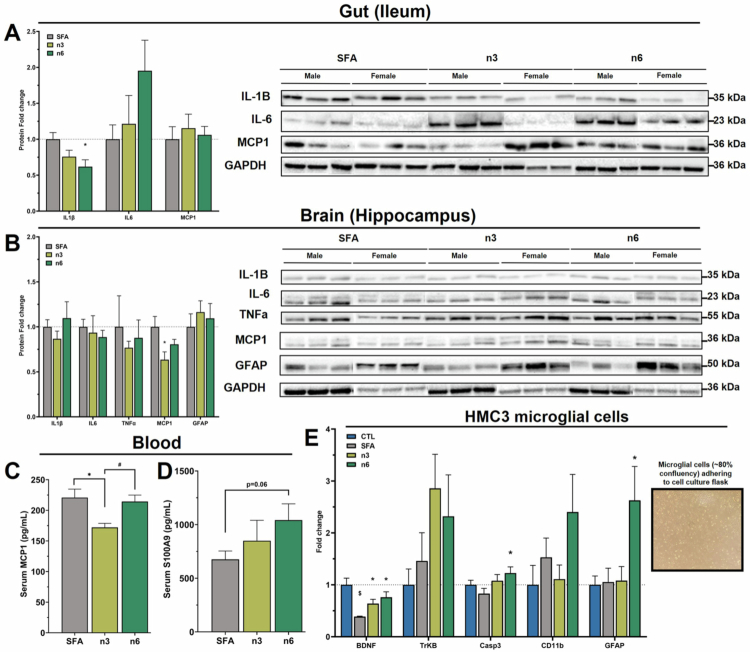
Maternal diet differing in fat sources differently shape the gut‒brain‒immune axis in neonates. Protein expression of inflammatory cytokines in the (A) ileum, (B) hippocampus, (C) serum MCP1 levels (pg/mL), (D) serum S100A9 levels (pg/mL), and (E) protein expression of BDNF signaling and microglial activation markers in HMC3 cells conditioned with mouse serum. Significance for protein expression analysis was calculated using the Kruskal–Wallis test with Dunn’s post-hoc analysis. ^$^ *p* < 0.05 between SFA and FBS-treated control groups, *p < 0.05 between SFA and PUFA groups, ^#^ *p* < 0.05 between n3 and n6 groups. The data are presented as mean ± SEM; *n* = 9–12 mice/group for panel A, *n* = 8–10 mice/group for panel B, *n* = 6–7 mice/group for panels C–D, and *n* = 5–6 mice/group for panel E.

Based on the observed potentiation of neuroinflammation by the n6 group and the counteracting effect of the n3 group compared to the SFA group, we further validated the immune profile in the ileum and hippocampus by assessing protein expression through Western blot (Figure 7A-B). Additionally, we performed ELISA to measure key pro-inflammatory markers in the serum (Figure 7C-D). Finally, the human microglial HMC3 cell line was exposed to mouse serum from these three groups to evaluate its response, focusing on BDNF signaling and microglial activation markers (Figure 7E). The ileal protein expression of IL1β was significantly reduced (*p* < 0.05) in the n6 group, while IL6 expression was relatively higher in the n6 group compared to the SFA group (Figure 7A). Additionally, the ileal protein expression of MCP1 remained unaffected overall, but its expression was significantly reduced (*p* < 0.05) in the n3 group when analyzed separately for males (Supplementary Figure S6). In the hippocampus, the expression levels of the MCP1 chemokine were also found significantly reduced (*p* < 0.05) for the n3 group compared to SFA group (Figure 7B). Consistent with these findings, the serum levels of MCP1 were significantly decreased (*p* < 0.05) in the n3 group compared to both the SFA and n6 groups (Figure 7C). In contrast, S1009, a subunit of calprotectin and a marker of pro-inflammation, was markedly increased (*p* = 0.06) in the n6 group (Figure 7D).

Finally, the human microglial cell line was treated with mouse serum to assess alterations in the gene expression of neuronal signaling and the inflammatory response (Figure 7F). The gene expression of BDNF in the HMC3 cell line was significantly reduced (*p* < 0.05) in the SFA-treated group compared to the CTL group (HMC3 cells treated with fetal bovine serum). Interestingly, BDNF levels were normalized in the n3- and n6-treated groups, which also showed an increase in TrkB expression. Additionally, the n6-treated group presented upregulated gene expression of pro-inflammatory markers such as Casp3, CD11b, and GFAP, indicating its pro-inflammatory role.

In addition, Spearman correlation analysis was performed to explore the association of MCP1 with microbial taxa, brain metabolites, inflammatory markers, and neurobehavioral parameters (Supplementary Figure S5). MCP1 demonstrated strong positive associations with *Coriobacteriaceae_UCG-002* and negative associations with *f_Lachnospiraceae;_* and *Parasutterella*, the former two taxa also being identified as discriminatory features through machine learning analysis. Furthermore, MCP1 was positively correlated with SFA-associated brain metabolites, including butanone, inosine, and succinic acid. Positive association trends were also observed between serum MCP1 and hippocampal MCP1 levels, supporting coordinated systemic and neuroinflammatory signaling. Importantly, MCP1 showed negative associations with neurobehavioral outcomes, including the T-maze alternation score and location memory discrimination index, while *Parasutterella* demonstrated a positive association with T-maze performance. Among the brain metabolites, serine was positively correlated with OPC and taurine, whereas sn-GPC was negatively correlated with serine and OPC. Collectively, these integrative associations support a potential relationship between microbiome-associated inflammatory signaling and neuro-metabolic outcomes.

### Maternal n3-rich diet preserves brain homeostasis via lipidome remodeling

**Figure 8.**
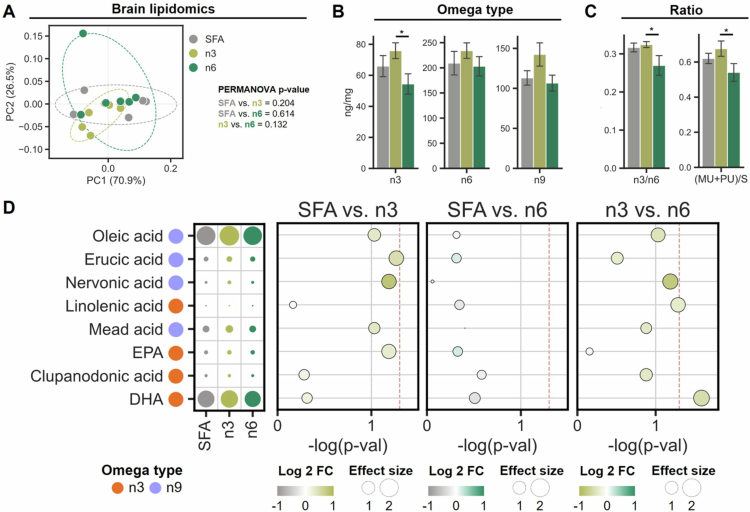
Maternal diet differing in fat sources differently shapes neonatal brain lipidomic profiles. (A) Beta-diversity of the brain lipidome as assessed using Bray-Curtis PCoA. (B) Abundance of fatty acids based on omega-type (ng/mg). (C) Abundance of fatty acids based on the n3/n6 ratio and the unsaturated/saturated fatty acid ratio. (D) Abundance and bubble plot for n3- and n9-type of fatty acids. The size of each bubble represents Cohen’s d effect size, and the color intensity indicates the log2-fold change between the two groups. Significance (*p* < 0.05) was calculated using the Mann‒Whitney U test and plotted as a -log (*p*-value) for each metabolite. The data are presented as mean ± SD; *n* = 6 mice/group.

Given that lipids are a major component of the brain, we sought to investigate whether maternal diets with varying levels of SFAs and PUFAs influence the brain lipidome of offspring at 8 weeks. Targeted lipidomics analysis of brain tissues identified 29 medium- to long-chain fatty acids, including 12 SFAs, 8 MUFAs, and 9 PUFAs. By chain length, 5 were medium-chain (C10–C15) fatty acids, and 23 were long-chain (C16–C24) fatty acids. Based on omega classification, 4 were n3 (linolenic acid, EPA, clupanodonic acid, and DHA), 2 were n5 (myristoleic acid and cis-10-pentadecenoic acid), 4 were n6 (linoleic acid, 11,14-eicosadienoic acid, AA, and adrenic acid), 3 were n7 (palmitoleic acid, cis-10-heptadecenoic acid, and 11-eicosenoic acid), and 4 were n9 (oleic acid, erucic acid, mead acid, and nervonic acid) (Figure 8 and Supplementary Figure S7). Although differences in lipid profiles analyzed using Bray–Curtis PCoA were not significant across the groups (Figure 8A); however, the n3 group exhibited significantly higher levels (*p* < 0.05) of n3-type fatty acids along with a significantly higher n3/n6 ratio, and PUFA/SFA ratio compared to the n6 group (Figure 8B and C).

Next, we examined how the concentration of key n3-type and n9-type fatty acids varied between groups using bubble plots (Figure 8D). Within the MUFA category, oleic and nervonic acids were appreciably enriched (*p* < 0.10) in the n3 group in comparison to both the SFA and n6 groups. Erucic acid exhibited a moderate increase (*p* < 0.10) in the n3 group relative to the SFA group. Notably, all these MUFAs demonstrated a relatively large effect size (*d* > 0.8), regardless of statistical significance. Within the n3-type category, EPA and mead acid were moderately enriched (*p* < 0.10) in the n3 group compared to the SFA group. Compared to the n6 group, DHA was significantly higher (*p* < 0.05) in the n3 group. These findings highlight the increased accretion of beneficial n3- and n9-type fatty acids in the brains of offspring exposed to an n3-rich maternal diet during periconception and perinatal periods, despite being exposed to an SFA-enriched diet post weaning.

## Discussion

This comprehensive study underscores the crucial role of the maternal diet during periconception, fetal development, birth, and weaning in conferring long-lasting benefits for offspring’s metabolic, immune, and neurocognitive health. Specifically, diets enriched with n3 PUFA and MUFA derived from fish and olive oils can protect offspring from the detrimental impacts of a Western diet rich in SFA during the periconceptional and perinatal periods. Our findings revealed that maternal diet significantly shapes the neonatal microbiome and metabolome at weaning, with specific features persisting into adulthood despite exposure to Western diet stressors. Further, we observed healthier features of the offspring’s neurocognitive and neurodevelopmental profiles, which are attributed to higher neuronal plasticity and lower neuroinflammation along with beneficial remodeling of the gut–brain–immune axis and brain lipidome.

Perinatal exposure to diets differing in fatty acid composition shapes offspring developmental trajectories and metabolism.^51^ We observed higher weight gain, though largely from increased muscle mass, and longer intestines and their villi in the n3 group, suggesting enriched nutrient absorption (Figures 2 and 5). Similar effects have previously been reported in swine, where maternal n3 fatty acid and hydroxytyrosol supplementation enhanced post-weaning growth and muscle mass;^52^ in contrast, we observed that the n6 group developed dyslipidemia, indicating abnormal metabolic imprinting (Figure 2). Excess arachidonic acid (AA) from n6 metabolism is known to promote adipogenesis via prostacyclin activation, potentially increasing fat mass and disrupting lipid metabolism when exposure is high during gestation and weaning.^53^ Adipogenesis outcomes also depend on timing: For instance, in young rodents, PUFAs have been found to reduce adipocyte number and size compared to SFAs, whereas perinatal PUFA exposure promotes adipocyte hyperplasia, and SFAs favor hypertrophy.^53^ These studies, along with our findings, cumulatively underscore the long-term metabolic imprinting of early-life fatty acid exposure.

Dietary fat intake influences cell signaling pathways and the progression of metabolic syndrome, with saturated fats increasing risk and certain unsaturated fats (e.g., fish oil and olive oil) offering protective effects.^54^ These effects are partly mediated through gut microbiome modulation, which regulates energy balance and inflammation, thereby shaping obesity and metabolic outcomes.^55^ We examined the transgenerational effects of the maternal diet on the neonatal gut microbiome and host health. Offsprings of PUFA-fed dams showed distinct microbial community shifts compared to those from SFA-enriched dams (Figure 3). These group-specific profiles, which were established at weaning, persisted into adulthood despite western-style diet feeding. While the PUFA groups had lower species richness, evenness remained stable, and the n3 neonates maintained distinct beta-diversity even after 8 weeks. *Erysipelotrichaceae* abundance was consistently higher in the n3 group at both time points, which is also consistent with the findings of a previous study.^54^ wherein olive oil (vs. butter) was found to promote this taxon. Although *Erysipelotrichaceae* members have been linked to lipid metabolism and inflammation, their exact role in metabolic disorders remains unclear.^56^ The n6 group showed higher *Blautia* and *Eubacterium_nodatum* *_group*. *Blautia* supports mucus homeostasis via FFAR2 activation from SCFA production (propionate, acetate),^57^ and is linked to exclusive breastfeeding and improved metabolic outcomes,^58^ though its role in obesity is unclear.^59^ *E. nodatum*, a periodontal pathogen, has been causally linked to obesity,^60^ and pro-inflammatory diets,^61^ as evident from its enrichment under n6 fatty acids, which can be pro-inflammatory in excess. Compared to SFA, the n6 group also presented a reduced abundance of *Parvibacter,* a genus promoted by prebiotics-rich diets and inversely associated with hepatic lipid levels,^62^ partly explaining their elevated lipids. In contrast, the n3 group exhibited lower abundance of *Butyricicoccaceae_UCG-009*, a taxon previously associated with increased weight gain and shorter colon length.^63^ This may partly align with the increased colon length observed in the n3 group, although causal interpretation requires further investigation (Figure 1).

Our untargeted global metabolomics analyses revealed distinct diet-induced effects on the gut, serum, and brain metabolite profiles. The n3 offsprings showed highly distinct fecal and brain metabolomes, while the n6 group had greater serum changes versus SFA (Figure 4). Offsprings from both PUFA groups presented increased levels of *N*-nitrosodimethylamine (NDMA) levels, a potentially carcinogenic compound formed from dietary or endogenous precursors. While it is technically challenging to determine the physiological levels of NDMA that could be detrimental in the present study, its presence has been attributed to precursors originating either exogenously from the diet or endogenously, such as heme iron, nitrates, nitrites, or excessive protein fermentation in the gut, which leads to the liberation of amines.^64^ Furthermore, the formation of nitroso compounds is influenced by stool transit time, with longer transit times increasing their abundance owing to enhanced microbiota-mediated biotransformation pathways.^65^ In offsprings from the n3 group, fecal succinate, a TCA cycle intermediate involved in gut–immune signaling via the SUCNR1 receptor, was uniquely higher. While excessive succinate accumulation has been associated with inflammatory responses, physiological levels of succinate have also been reported to promote muscle protein deposition, reduce adiposity, and improve insulin sensitivity.^66^ Therefore, the higher succinate levels observed in the n3 offspring may potentially be associated with the increased lean mass phenotype observed in this group. However, this interpretation is based on previously reported literature and should be interpreted cautiously within the context of the present dataset, as the current study does not establish a direct mechanistic relationship. Future studies employing targeted mechanistic approaches should aim to clarify this association. We also observed higher levels of isoleucine in both the fecal and serum niches in the n6 group, a pattern that has previously been linked to accelerated metabolic syndrome and neurotoxic disorders in high-fat diet models, potentially contributing to the observed neuroinflammatory phenotype.^67^ The n6 group also presented increased ethanol, acetate, citric acid, and creatine levels, indicating disruption of the mitochondrial and TCA cycles. Elevated acetate and ethanol have been found to be associated with obesity, type-2 diabetes, and gut dysbiosis phenotypes,^68^ ^,^ ^69^ while high plasma citrate has mixed implications linked to macrophage–monocyte-driven inflammation,^70^ and improved physical performance.^71^ Thus, it should be an interesting subject for future studies to determine whether and how elevated citrate levels reflect a compensatory mechanism against metabolic disturbances or simply more active cellular metabolism. Our findings of lower serum taurine in n6 group further reflect a pro-inflammatory milieu, as taurine functions as an antioxidant and protects immune cells from oxidative stress.^72^ Our brain metabolomics data revealed that both PUFA groups had higher serine, a metabolite essential for neuronal differentiation and survival.^73^ L-serine, produced by astrocytes from glucose, is a precursor for neuronal D-serine, which co-activates NMDA receptors with glutamate to support memory and cognition. Lower brain serine levels have been reported in high-fat diet-fed mice and are associated with cognitive and metabolic deficits.^74^ ^,^ ^75^ We also note that the offspring from n3-fed dams presented uniquely higher OPC and lower snGPC, indicating enhanced phosphatidylcholine synthesis and membrane stability.^76^ In contrast, the SFA group presented higher levels of metabolites (e.g., creatine phosphate, inosine) that have been associated with acute stress responses,^77^ and elevated snGPC, which are also linked to enhanced phosphatidylcholine hydrolysis observed in Alzheimer’s pathology.^78^ Taken together, these findings suggest how maternal and prenatal n3 PUFAs intake favors a healthier gut and brain development, while n6 PUFAs may induce serum metabolomic signatures indicative of impaired or dysregulated metabolic function.

Mucosal homeostasis is essential for the controlled regulation of epithelial integrity and immune function. Disruption of this niche through hyper-permeability (“gut leakiness”) or hyper-inflammation can drive local and systemic disorders. We observed that the maternal n3 supplementation improved neonatal intestinal barrier function, while both PUFA groups presented downregulated pore-forming claudins (CLDN2, 12, 15) with distinct modulations in tight-junction scaffolding proteins (ZO1, OCCL, JAM3) (Figure 5). CLDN2 upregulation is typically a marker of leaky gut (hyperpermeability),^79^ while CLDN12 and CLDN15, though linked to cation transport, may also influence permeability and epithelial proliferation.^80^ ^,^ ^81^ Dysregulated expression of these proteins corresponded with observed group-specific changes in mucosal architecture. Interestingly, despite the reduced ileal mRNA expression of ZO1, OCCL, and JAM3 in the n3 and n6 groups, these changes may not necessarily indicate impaired epithelial barrier integrity. Emerging evidence from knockout animal models suggests that basal intestinal barrier function can remain preserved even in the absence of OCCL or ZO1 under physiological conditions, highlighting additional non-canonical roles of these proteins in epithelial proliferation, apoptosis, and tissue remodeling.^82^ Likewise, JAM3 participates in epithelial, endothelial, and immune signaling pathways, and its reduced expression has also been associated with altered epithelial turnover and mucosal immune activation.^83^ Importantly, the n3 group simultaneously exhibited lower inflammatory signatures and beneficial microbiome-associated alterations, suggesting that transcriptional modulation of selected junction-associated genes may reflect dietary PUFA-mediated epithelial remodeling rather than overt barrier dysfunction. Nevertheless, these findings should be interpreted cautiously, as mRNA expression alone may not directly reflect functional tight-junction integrity. Future studies involving protein-level validation and immunohistological localization will be necessary to clarify the mechanistic significance of these observations during early-life developmental programming. Offspring from n3-fed dams presented selective upregulation of FFAR2 and MCT1, indicating a synergy between n3 PUFAs and SCFAs metabolism. Propionate and acetate, potent FFAR2 agonists, are known to enhance antimicrobial defense, immune differentiation, and satiety.^84^ ^,^ ^85^ We also observed elevated levels of fecal succinate, a propionate precursor, in n3 offspring, which may underlie the observed upregulated FFAR2 expression. The longer villi and higher villus length/crypt depth ratios observed in this group further reflect healthier enterocytes, improved absorptive areas, and enriched nutrient uptake, which is consistent with findings from maternal DHA supplementation studies.^86^ ^,^ ^87^

We also found that a maternal PUFA-rich diet led to significantly upregulated expression of markers related to synaptic plasticity, neurogenesis, and long-term potentiation in the offspring, indicating transgenerational benefits in neuronal function (Figure 6). Enhanced BDNF/TrKB/CREB signaling suggests improved neuronal plasticity, impairments of which have been linked to Alzheimer’s disease.^88^ Enhanced hippocampal BDNF signaling has been reported in schizophrenia models.^89^ Increased DHA accretion and neurotrophin expression in the cortex following perinatal and post-weaning n3 diets have also been reported.^90^ PSD95, a scaffolding protein essential for synaptic strength, is known to be downregulated by high-fat diets and early-life stress.^91^ Maternal supplementation of n3 fatty acids has also previously been associated with enhanced intelligence and physical coordination in infants.^92^ Our findings suggest that maternal n3 supplementation delivered transgenerational benefits, eliciting favorable impacts on short-term working memory, as assessed via T-maze alteration, though the literature reports mixed results,^93-95^ which might be attributed to differences in study design, dosing, timing, spontaneous or reward-driven alteration, and species. We observed significant variation in neuroinflammatory outcomes between the groups, with mitigated inflammation in the n3 group but exacerbated inflammation in the n6 group. Hippocampal upregulation of the microglial markers CD16 (M1) and CD206 (M2) seen in the n6 group suggests a transitional activation state, although higher IL6 and MCP1 regulation indicated a predominantly pro-inflammatory phenotype.^96^ ^,^ ^97^ Conversely, the n3 group has suppressed GFAP, CD11b, and Casp1 regulation, which is consistent with earlier studies reporting that n3 diets inhibit inflammasome activation and glial reactivity.^98^ ^,^ ^99^ Based on our findings, MCP1 has emerged as a key gut‒blood‒brain axis mediator that is downregulated in n3 but elevated in n6, potentially linking gut microbiota-driven peripheral inflammation to neuroimmune dysfunction (Figure 7). MCP1, a chemokine facilitating monocyte migration in inflamed environments,^100^ has been linked to elevated serum levels in metabolic syndrome.^101^ Its ability of MCP1 to cross the intact blood‒brain barrier from the circulation further exacerbates neuroimmune dysfunction under systemic inflammation.^102^ Our subsequent analyses testing the peripheral effects of blood on the inflammatory profile of human microglial cells further corroborated the observed association between PUFA-driven restoration of BDNF signaling and the n6-induced pro-inflammatory milieu.

Our further analyses providing insights into brain lipidomics revealed higher beneficial PUFAs, particularly n3 and n9 types, in offspring from n3-fed dams (Figure 8). Oleic acid, known to be linked to better neurocognitive function, reduced cognitive decline, and is central to Mediterranean and Okinawan diets, has anti-inflammatory and neuroprotective roles.^103^ ^,^ ^104^ Our findings of elevated oleic acid in n3 offspring concur with elevated nervonic, mead, and erucic acids, which are critical for myelin formation, brain development, and cognitive function.^105^ Nervonic acid deficiency is known to be associated with impaired infant brain growth,^106^ while erucic acid has been known to enhance memory-related signaling and reduce neuroinflammation.^107^ We find that, as compared to offsprings from n6-fed dams, those from n3 offsprings had enriched palmitoleic, linoleic, linolenic, adrenic, and DHA levels, all of which are known to confer anti-inflammatory effects, including the inhibition of palmitic acid-induced microglial activation.^108^ ^,^ ^109^ A higher n3/n6 ratio in the n3 group supports improved brain development, likely due to placental transfer of EPA and DHA in late gestation. This is consistent with previous studies linking a higher omega-3 index to larger brain volumes, enhanced cognition, and reduced neurological disease risk.^110^ To this end, our findings highlight the significant neonatal benefits of n3-enriched prenatal and perinatal nutrition and corroborate previous reports suggesting its lasting effects on brain DHA levels and synaptic markers such as PSD95.^111^

The present study has several limitations that should be acknowledged. A major limitation is the inability to establish a direct causal role of the gut microbiome in mediating the observed metabolic and neurodevelopmental effects. While the study identified significant links between maternal diet, microbiome composition, metabolomic alterations, and host phenotypes, definitive validation of microbiome-mediated mechanisms would require targeted causal approaches such as fecal microbiota transplantation, antibiotic depletion, or monocolonization experiments in germ-free or gnotobiotic models. In addition, the inclusion of chow-fed offspring controls and cross-fostering experiments would further strengthen the mechanistic interpretation by helping distinguish prenatal effects from lactational and postnatal influences. The current study also included limited temporal sampling of the offspring microbiome; therefore, future longitudinal studies incorporating more frequent sampling during lactation, weaning, and post-weaning periods will be helpful to better delineate the timing and persistence of maternal diet-associated microbiome programming effects. Furthermore, metabolomic analyses were performed by standard procedures using whole-brain homogenates, but these do not capture region-specific metabolic heterogeneity. Future studies involving targeted metabolomics, lipidomics, and transcriptomics analyses of specific brain regions, such as the hippocampus, cortex, and hypothalamus, may help provide deeper mechanistic insight into the neurodevelopmental and neurometabolic programming associated with maternal dietary fatty acid exposure.

## Conclusion

The findings from this inclusive and multi-omics study reveal profound impacts of maternal nutrition on long-lasting transgenerational health outcomes in the offspring. The data evince how exposure to an n3 fatty acid-enriched diet during gestation and lactation shapes developmental programming, protecting offspring from the adverse gut, metabolic, and neurocognitive effects due to Western-style diet exposure during post-weaning periods into adulthood. This transgenerational protection entails persistent microbiome and metabolome remodeling and homeostatic regulation of neurodevelopment via active phosphatidylcholine synthesis, upregulated BDNF/TrkB signaling, controlled neuroinflammation, and brain retention of key MUFAs and n3 PUFAs. Downstream assessment of inflammation origins underscores the contribution of a gut–brain–immune axis mechanism in brain homeostasis, with chronic inflammation mediated by MCP1 being tightly controlled and suppressed by a maternal n3 diet. Taken together, these findings highlight the importance of n3-enriched maternal diets during critical periconceptional and perinatal windows in promoting healthy neonatal ontogenesis while mitigating the risk of chronic diseases due to imprudent dietary exposures during prenatal or postnatal life stages.

## Supplementary Material

Supplementary Material Supplementary_Data_MS_264793374_R1 clean.docx

## Data Availability

All datasets generated for this study are included in the article/Supplementary material. All the raw sequencing datasets have been submitted to the NCBI Sequence Read Archive (SRA) public repository database under SRA BioProject number PRJNA1258582.
