# Supplementary material for: Maternal diet shapes neonatal microbiome ontogenesis and neurometabolic resilience
Source: Gut Microbes. 2026 Jun 9;18(1):2684074. doi: 10.1080/19490976.2026.2684074 (PMC13251521; doi:10.1080/19490976.2026.2684074)
Supplement: Supplementary Material — Supplementary_Data_MS_264793374_R1 clean.docx [file KGMI_A_2684074_SM2477.docx]

**Maternal diet shapes neonatal microbiome ontogenesis and neurometabolic resilience**

Saurabh Kadyan^1,2,3^, Gwoncheol Park^1,2^, Leila Khalili^2^, Cole Patoine^1,2^, Moses Mayonu^4^, Bo Wang^4^, Gloria Salazar^2^, Yuichiro Yamashiro^5^, Ravinder Nagpal^1,2,6^*

^1^The Gut Biome Lab, Florida State University, Tallahassee, Florida 32306, USA.

^2^Department of Health, Nutrition, and Food Sciences, Florida State University, Tallahassee, Florida 32306, USA.

^3^Dairy Microbiology Division, National Dairy Research Institute, Karnal, Haryana 132001, India.

^4^College of Engineering and Science: Chemistry and Chemical Engineering, Florida Institute of Technology, Melbourne, Florida 32901, USA.

^5^Probiotics Research Laboratory, Juntendo University School of Medicine, Bunkyo City, Tokyo 1138421, Japan.

^6^Center for Integrative Nutrition and Food Research, Florida State University, Tallahassee, Florida 32306, USA.

*Correspondence: [rnagpal@fsu.edu](mailto:rnagpal@fsu.edu)

**Table S1. Diet composition used in the study (customized through ResearchDiets Inc, USA)**

|  | **WD** | **SFA** | **N6** | **N3** |
| --- | --- | --- | --- | --- |
| Cat # | D22071301 | D22071302 | D22071303 | D22071304 |
| Ingredient | gm | gm | gm | gm |
| Casein | 38.5 | 38.5 | 38.5 | 38.5 |
| Fish Protein Isolate | 8.5 | 8.5 | 8.5 | 8.5 |
| Egg white | 55 | 55 | 55 | 55 |
| Beef, cooked, powdered | 76.9 | 76.9 | 76.9 | 76.9 |
| L-cystine | 3 | 3 | 3 | 3 |
| Corn starch | 15 | 15 | 15 | 15 |
| Wheat starch | 165.5 | 165.5 | 165.5 | 165.5 |
| Potato Starch | 15 | 15 | 15 | 15 |
| Sucrose | 205 | 205 | 205 | 205 |
| Fructose | 22 | 22 | 22 | 22 |
| Cellulose | 18.2 | 18.2 | 18.2 | 18.2 |
| Inulin | 6 | 6 | 6 | 6 |
| Menhaden (Fish) oil | 1.2 | 0 | 0 | 13.7 |
| Corn Oil | 0 | 0 | 140.7 | 0 |
| Palm Kernel Oil | 0 | 0 | 0 | 0 |
| Butter, anhydrous | 64 | 140.7 | 0 | 0 |
| Lard | 41.3 | 0 | 0 | 0 |
| Flaxseed oil | 1 | 1.2 | 0 | 0 |
| Olive oil | 33 | 0 | 0 | 127 |
| t-BHQ | 0.0047 | 0.0047 | 0.0047 | 0.0047 |
| Mineral mix | 10 | 10 | 10 | 10 |
| Dicalcium phosphate | 13 | 13 | 13 | 13 |
| Calcium carbonate | 5.5 | 5.5 | 5.5 | 5.5 |
| Potassium Citrate | 16.5 | 16.5 | 16.5 | 16.5 |
| Vitamin mix | 10 | 10 | 10 | 10 |
| Biotin | 0.1 | 0.1 | 0.1 | 0.1 |
| Choline Bitartrate | 2 | 2 | 2 | 2 |
| Cholesterol | 1.5 | 1.5 | 1.5 | 1.5 |
| Resveratrol (50% Trans) | 0 | 0 | 0 | 0 |
| **Gm%** |  |  |  |  |
| Protein | 16.5 | 16.5 | 16.5 | 16.5 |
| Carbohydrate | 48.9 | 48.9 | 48.9 | 48.9 |
| Fat | 20 | 20 | 20 | 20 |
| Fiber | 2.9 | 2.9 | 2.9 | 2.9 |
| **kcal** |  |  |  |  |
| Protein | 547 | 547 | 547 | 547 |
| Carbohydrate | 1620 | 1620 | 1620 | 1620 |
| Fat | 1493 | 1493 | 1493 | 1493 |
| Total | 3660 | 3660 | 3660 | 3660 |
| **Kcal%** |  |  |  |  |
| Protein | 15 | 15 | 15 | 15 |
| Carbohydrate | 44 | 44 | 44 | 44 |
| Fat | 41 | 41 | 41 | 41 |
| Kcal/g | 4.42 | 4.42 | 4.42 | 4.42 |
| n6:n3 ratio^#^ | 6.0 | 1.6 | 42.9 | 3.3 |

#Calculated based on the ingredient composition of fat sources only.

**Table S2. List of primer sequences used in the study**

| **Gene name** | **Forward primer** | **Reverse primer** |
| --- | --- | --- |
| 16S | GTGYCAGCMGCCGCGGTAA | GGACTACNVGGGTWTCTAAT |
| 18S | AGAAACGGCTACCACATCCA | CCCTCCAATGGATCCTCGTT |
| CLDN1 | GGCTTCTCTGGGATGGATCG | CTTTGCGAAACGCAGGACAT |
| CLDN2 | CCGTGTTCTGCCAGGATTCTC | AGGAACCAGCGGCGAGTAG |
| CLDN5 | GTTAAGGCACGGGTAGCACT | TACTTCTGTGACACCGGCAC |
| CLDN12 | GAGCCGATGTGCTCCTGTT | GGAGGGCTTGAGCTGTATGG |
| CLDN15 | AGGCACACCTTATCTGGCAC | TGCCCCCTGAACAATCACAA |
| ZO1 | AAGAAAAAGAATGCACAGAGTTGTT | GAAATCGTGCTGATGTGCCA |
| ZO2 | AGCTTGTAGTTCTGAGCCGC | CCGACACGGCAATTCCAAAT |
| OCCL | CTGACTATGCGGAAAGAGTTGAC | CTGACTATGCGGAAAGAGTTGAC |
| JAM3 | GCTGTGAGGTCGTTGCTCTA | AGTGGCACATCATTGCGGTA |
| IL1β | GAAATGCCACCTTTTGACAGTG | TGGATGCTCTCATCAGGACAG |
| IL6 | GTCCTTCCTACCCCAATTTCCA | CGCACTAGGTTTGCCGAGTA |
| IL8 | ACTCAAGAATGGTCGCGAGG | GTGCCATCAGAGCAGTCTGT |
| IL10 | TGGGTTGCCAAGCCTTATCG | TTCAGCTTCTCACCCAGGGA |
| IL17A | TCCCTCTGTGATCTGGGAAG | CTCGACCCTGAAAGTGAAGG |
| TNFα | GATCGGTCCCCAAAGGGATG | TTTGCTACGACGTGGGCTAC |
| MCP1 | CCCAATGAGTAGGCTGGAGA | TCTGGACCCATTCCTTCTTG |
| BDNF | TCATACTTCGGTTGCATGAAGG | AGACCTCTCGAACCTGCCC |
| PSD-95 | ACGCCGAAGAGTCAGAGAAA | ACTGTTGGACCGAGTGAACC |
| DCX | GATGTCAACCGGGAAAGCAC | GTGGAACCACAGCAACTTTTC |
| CD11b | AATGATGCTTACCTGGGTTAT GCT | TGATACCGAGGTGCTCCTAAAAC |
| Casp1 | GGACATCCTTCATCCTCAGAAACA | TTTCTTTCCATAACTTCTGGGCTTT |
| Egr-1 | AGCGAACAACCCTATGAGCA | ATAACTCGTCTCCACCATCCGC |
| GFAP | ACGATCTGGCTTGGTCATTCTGG | AGGTTCCCTGAGCATGTCTGCTT |
| CD16 | TTTGGACACCCAGATGTTTCAG | GTCTTCCTTGAGCACCTGGATC |
| CD206 | GGTTCCGGTTTGTGGAGCAG | TCCGTTTGCATTGCCCAGTA |
| TrKB | CGGCACATAAATTTCACACG | TTACCCGTCAGGATCAGGTC |
| CREB | ACAATGGTACGGATGGGGTA | CTGCTGTCCATCAGTGGTC |
| FFAR2 | ATCCTCACGGCCTACATCCT | CAGCAGCAACAACAGCAAGT |
| FFAR4 | CCATCCCTCTAGTGCTCGTC | TGCGGAAGAGTCGGTAGTCT |
| MCT1 | GGTGTCATTGGAGGTCTTGGG | GGCCAATGGTCGCTTCTTG |
| SMCT1 | CTGGGCTTGTTTTCTTTGG | CGTTGTGCGTGCTGTTAC |
| h-BDNF | GGCTTGACATCATTGGCTGAC | CATTGGGCCGAACTTTCTGGT |
| h-TrkB | TCGTGGCATTTCCGAGATTGG | TCGTCAGTTTGTTTCGGGTAAA |
| h-Casp3 | CATGGAAGCGAATCAATGGACT | CTGTACCAGACCGAGATGTCA |
| h-CD11b | ACTTGCAGTGAGAACACGTATG | TCATCCGCCGAAAGTCATGTG |
| h-GFAP | CTGCGGCTCGATCAACTCA | TCCAGCGACTCAATCTTCCTC |
| h-GAPDH | AAGGTCGGAGTCAACGGATT | AAGGTCGGAGTCAACGGATT |

**Table S3: Duration of dietary exposure from breeding to delivery and cumulative exposure during gestation and lactation**

| **Group*** | **Exposure duration from breeding to delivery (days; mean ± SD)** | **Total exposure duration until weaning (days; mean ± SD)** |
| --- | --- | --- |
| **SFA** | 22.67 ± 3.18^a^ | 43.67 ± 3.18 ^A^ |
| **n3** | 23.39 ± 4.50 ^a^ | 44.39 ± 4.50 ^A^ |
| **n6** | 23.78 ± 2.83 ^a^ | 44.78 ± 2.83 ^A^ |

*Three breeder pairs per group

Data are presented as mean ± SD

Values sharing the same superscript letter within a column are not significantly different at *p* < 0.05

**Table S4: Litter-wise distribution of mice across groups**

| **Group** | **Total mice per group** | **Mice in litter-1 (M/F)** | **Mice in litter-2 (M/F)** | **Mice in litter-3 (M/F)** |
| --- | --- | --- | --- | --- |
| **SFA** | 17 | 5(2/3) | 6 (3/3) | 6 (3/3) |
| **n3** | 16 | 5 (2/3) | 6 (3/3) | 5 (2/3) |
| **n6** | 20 | 7 (3/4)) | 6 (3/3) | 5 (2/3) |

M = males; F = females


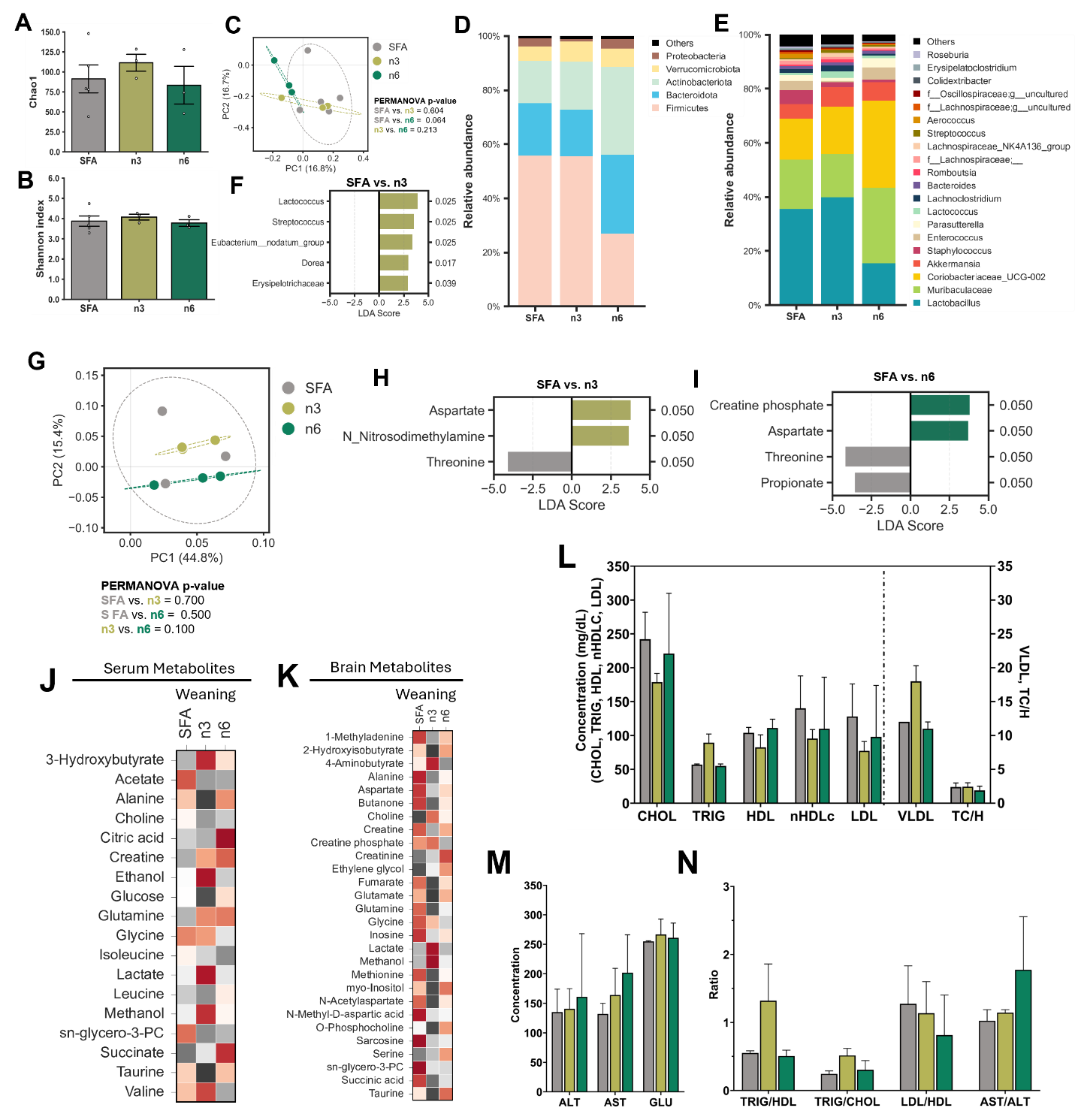


**Figure S1. Microbiome, metabolome, and lipid profiling of breeder dams (A)** Alpha-diversity assessed using **(A)** the Chao1 index and **(B)** the Shannon index. **(C)** Microbiome beta-diversity assessed using Bray-Curtis PCoA analysis, with significance determined by PERMANOVA (p < 0.05). Taxonomic relative abundance at **(D)** Phylum level and **(E)** Genus level. **(F)** Linear discriminant analysis effect size (LEfSe) with an LDA score ≥ 2.0 and p < 0.05 showing group-specific discriminatory taxa. **(G)** Fecal metabolome beta-diversity assessed using Bray-Curtis PCoA analysis, with significance determined by PERMANOVA (p < 0.05). Linear discriminant analysis effect size (LEfSe) with an LDA score ≥ 2.0 and p < 0.05 showing group-specific discriminatory taxa between **(H)** SFA vs n3 groups, and **(I)** SFA vs n6 groups. **(J)** Heatmap showing relative abundance of serum metabolites among groups for weaned pups. **(K)** Heatmap showing relative abundance of brain metabolites among groups for weaned pups. Color shade from red to dark grey indicates gradient of decreasing relative abundance. **(L)** Circulating lipoproteins concentration (mg/dL) of dams **(M)** Hepatic markers and glucose concentration (mg/dL) of dams. **(N)** Lipid ratios of dams. CHOL: Cholesterol; TRIG: Triglycerides; HDL: High-density lipoprotein cholesterol; LDL: Low-density lipoprotein cholesterol; VLDL: Very-low-density lipoprotein cholesterol; TC/H: Total cholesterol to HDL ratio; ALT: Alanine aminotransferase; AST: Aspartate aminotransferase; GLU: Glucose. Data are presented as mean ± SEM; n = 3-5 mice/group for panel A-F, n = 3 mice/group for panel G-I, n = 1-8 mice/group for panel J-K and n = 2-3 mice/group for panels L-N.


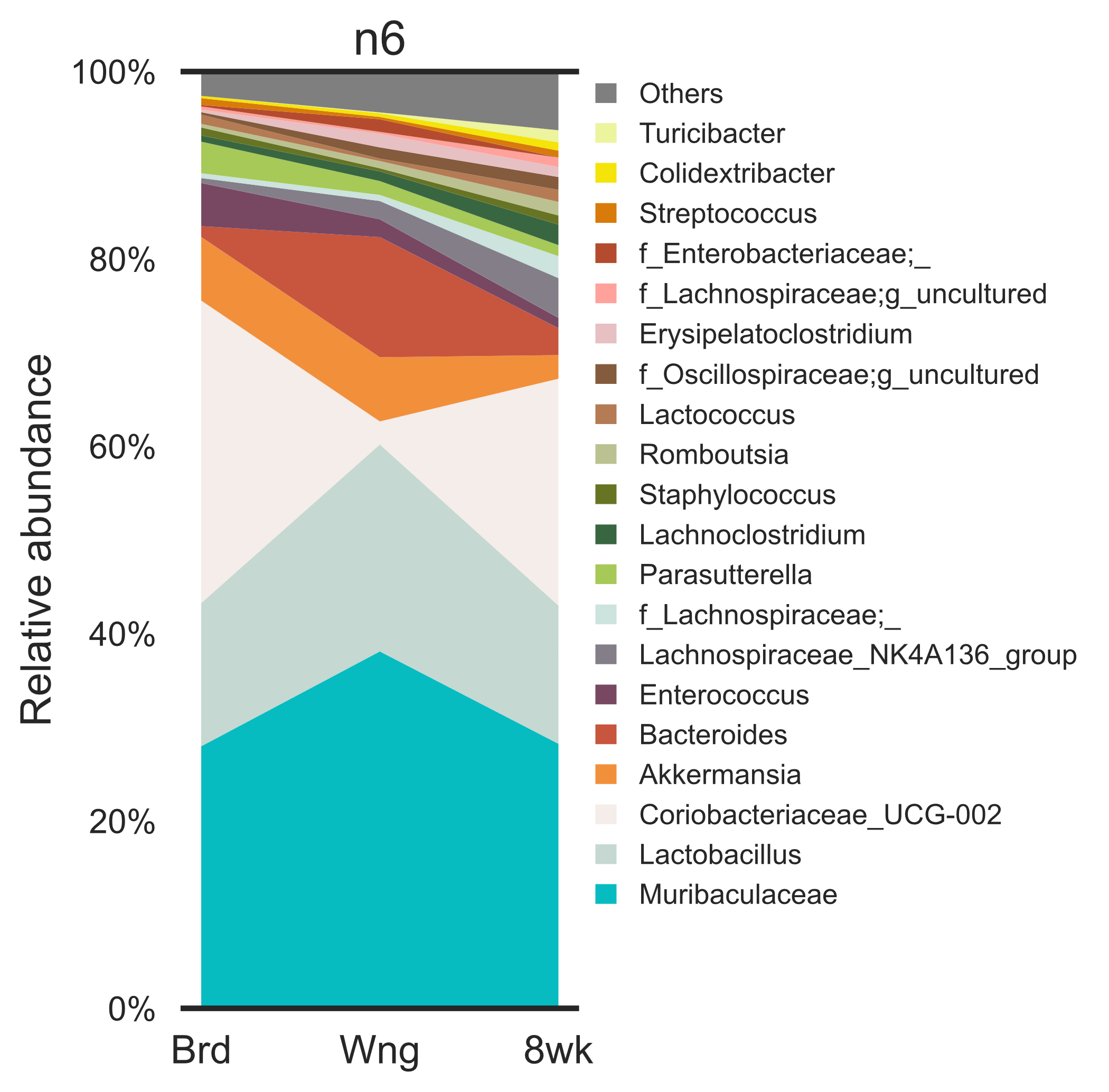

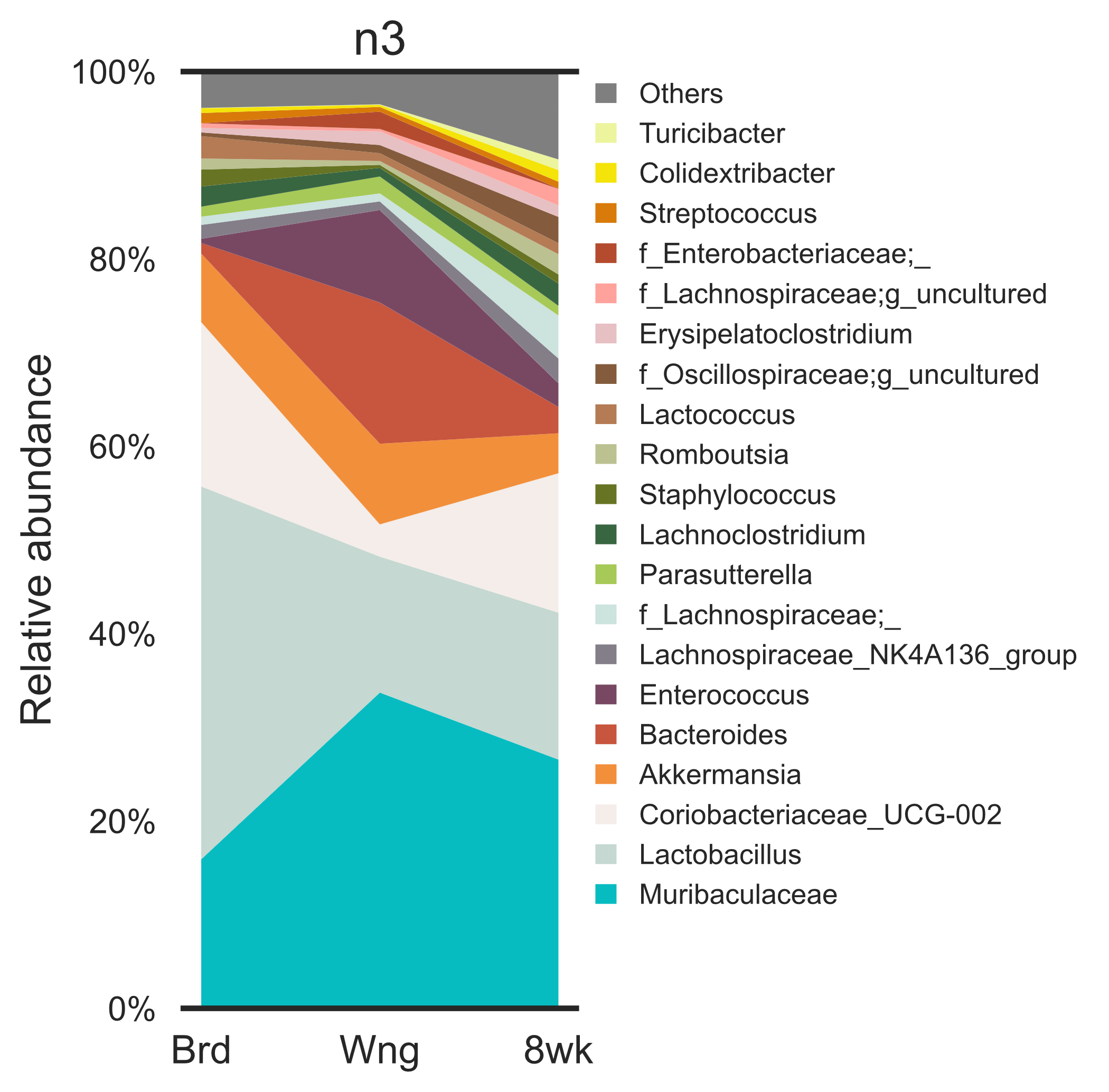

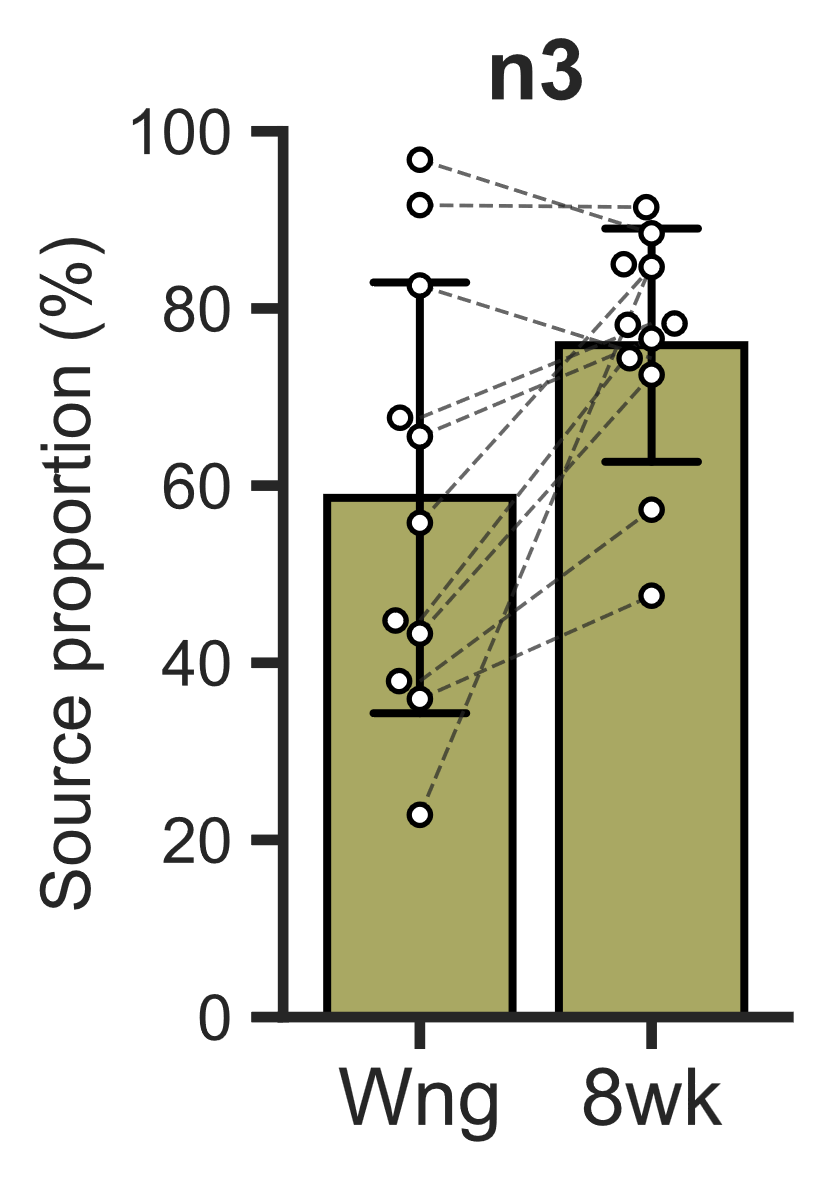

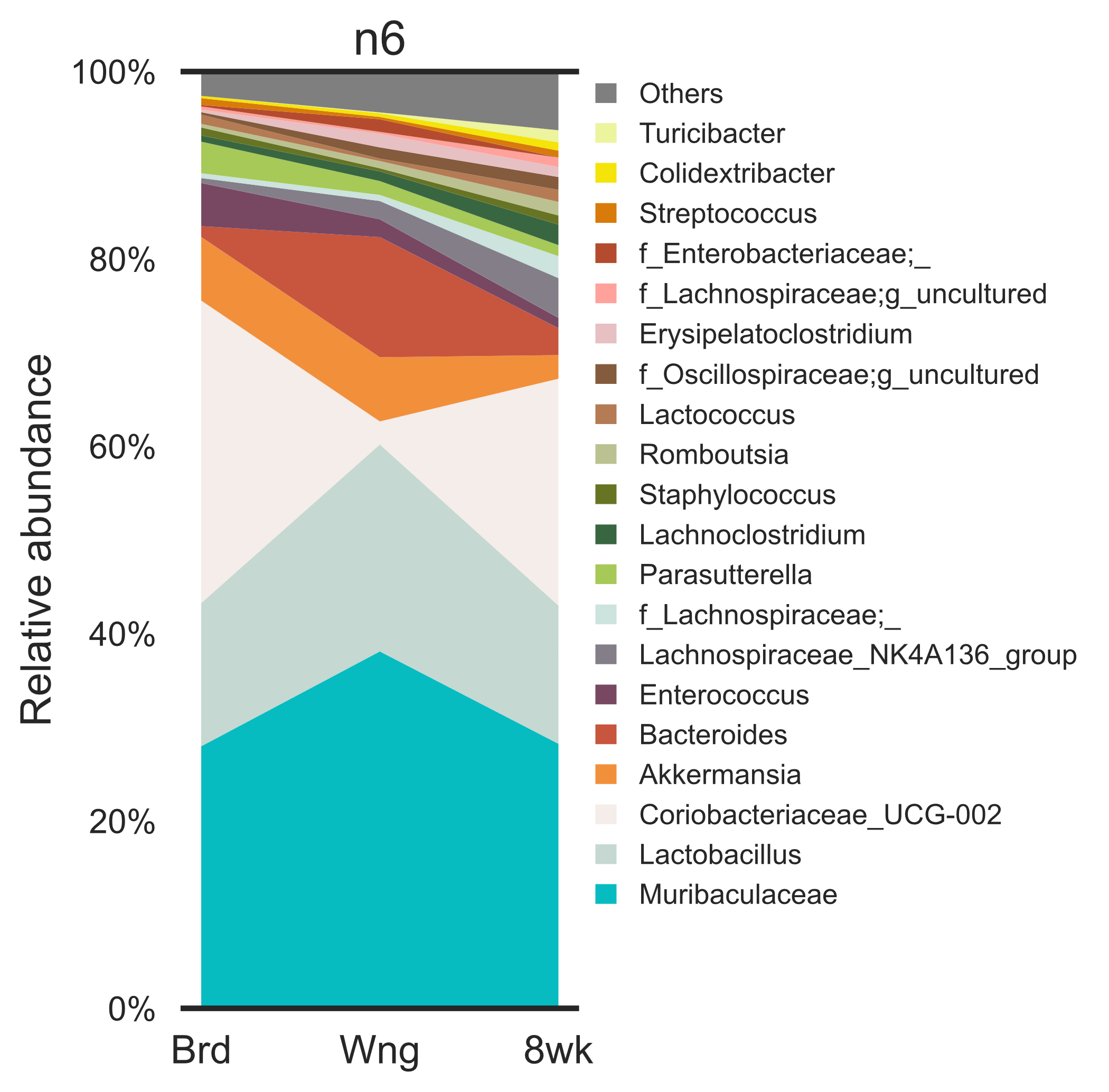

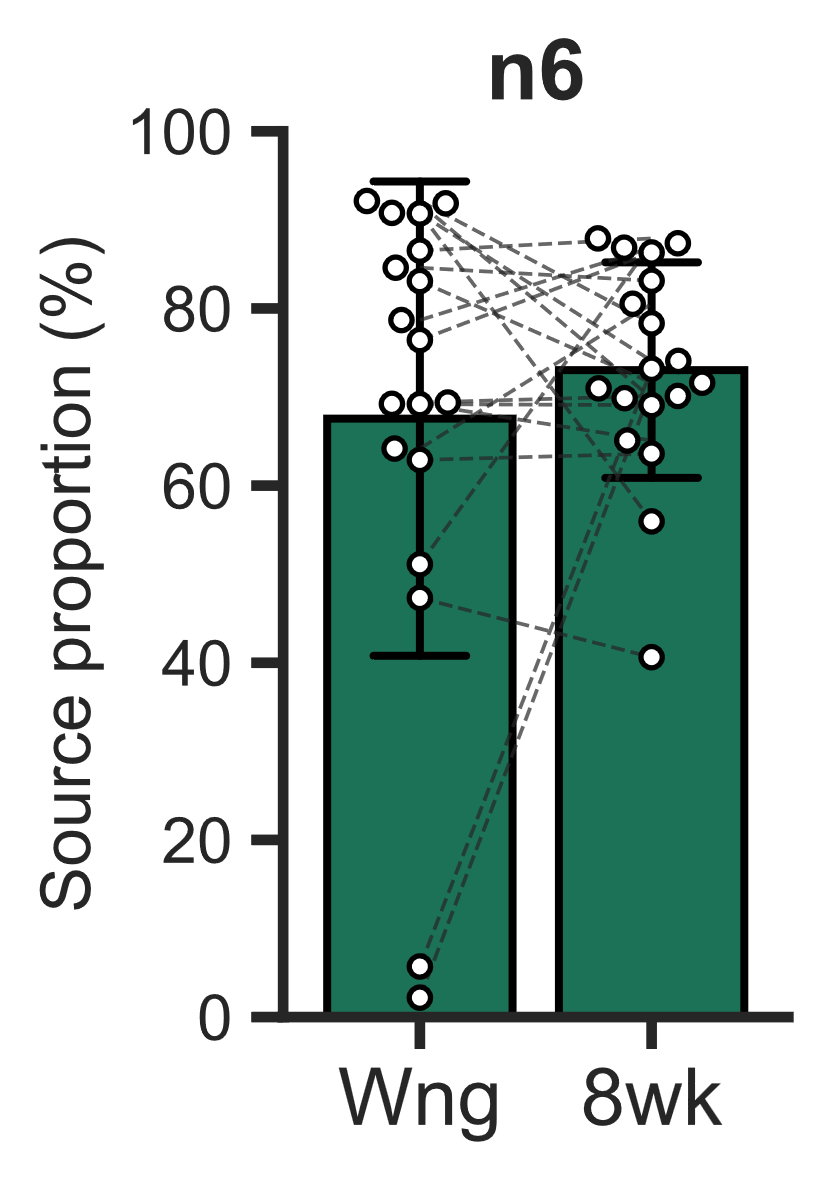

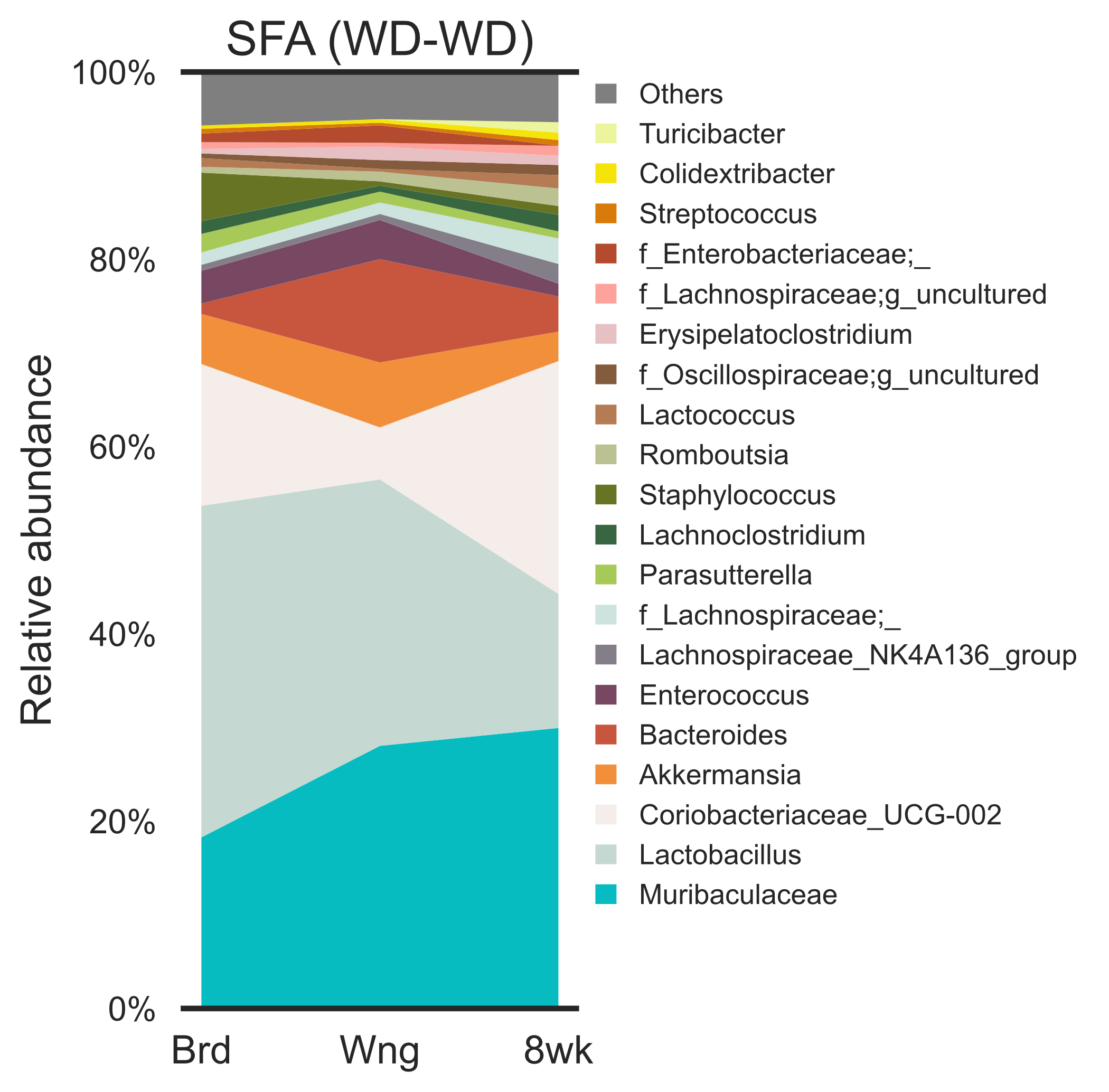

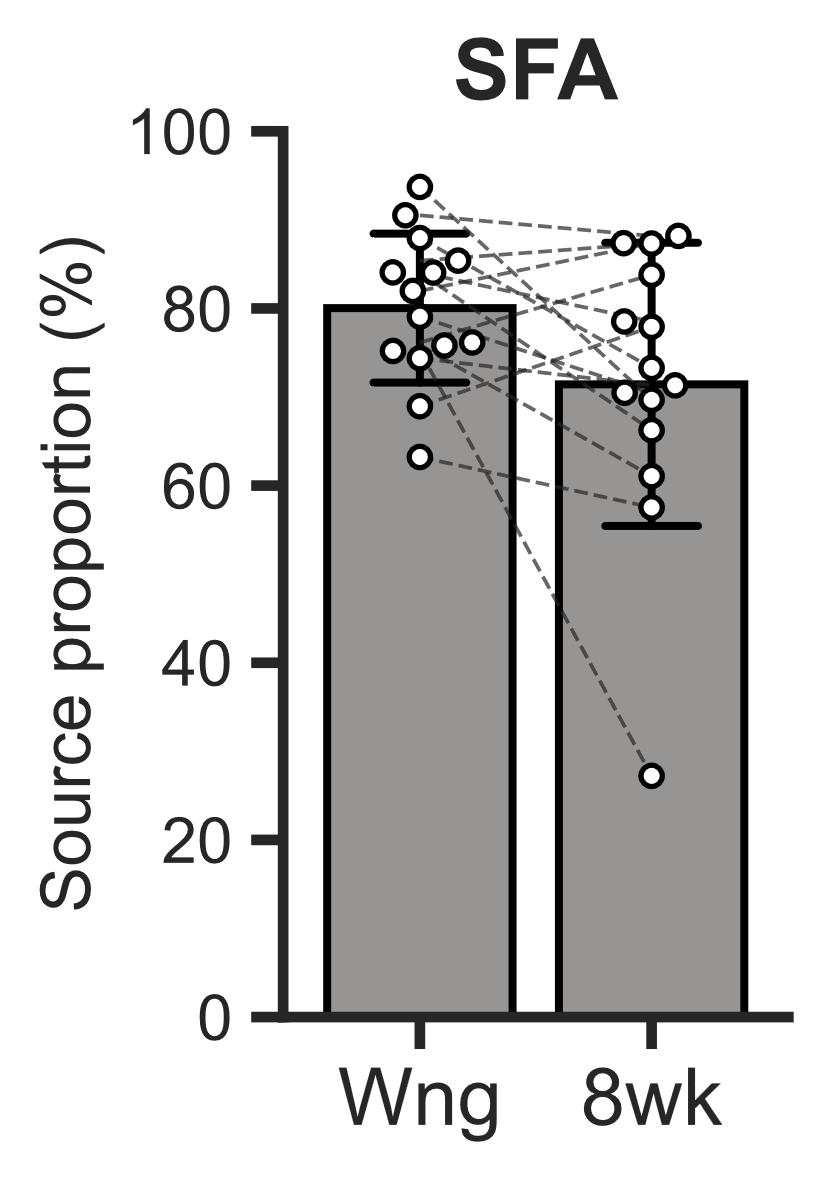


**A**

**B**

**C**

**Figure S2. Temporal changes in relative abundance of gut microbiome and proportion of the gut microbiome transmitted from breeders to their offspring and maintained up to 8-weeks of age** for (A) SFA group, (B) n3 group, and (C) n6 group. Data are presented as mean ± SD; n = 11-18 mice/group.


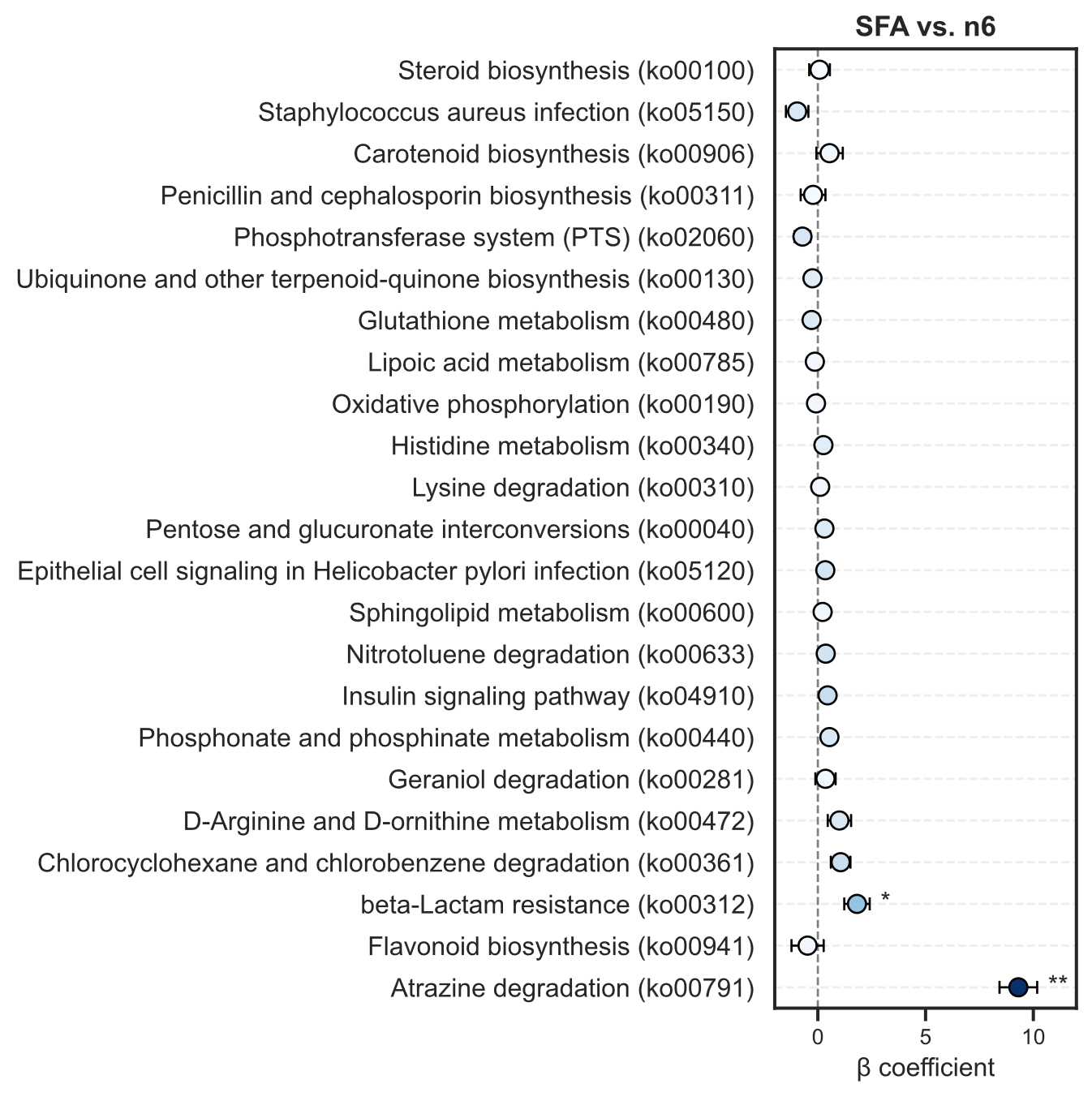

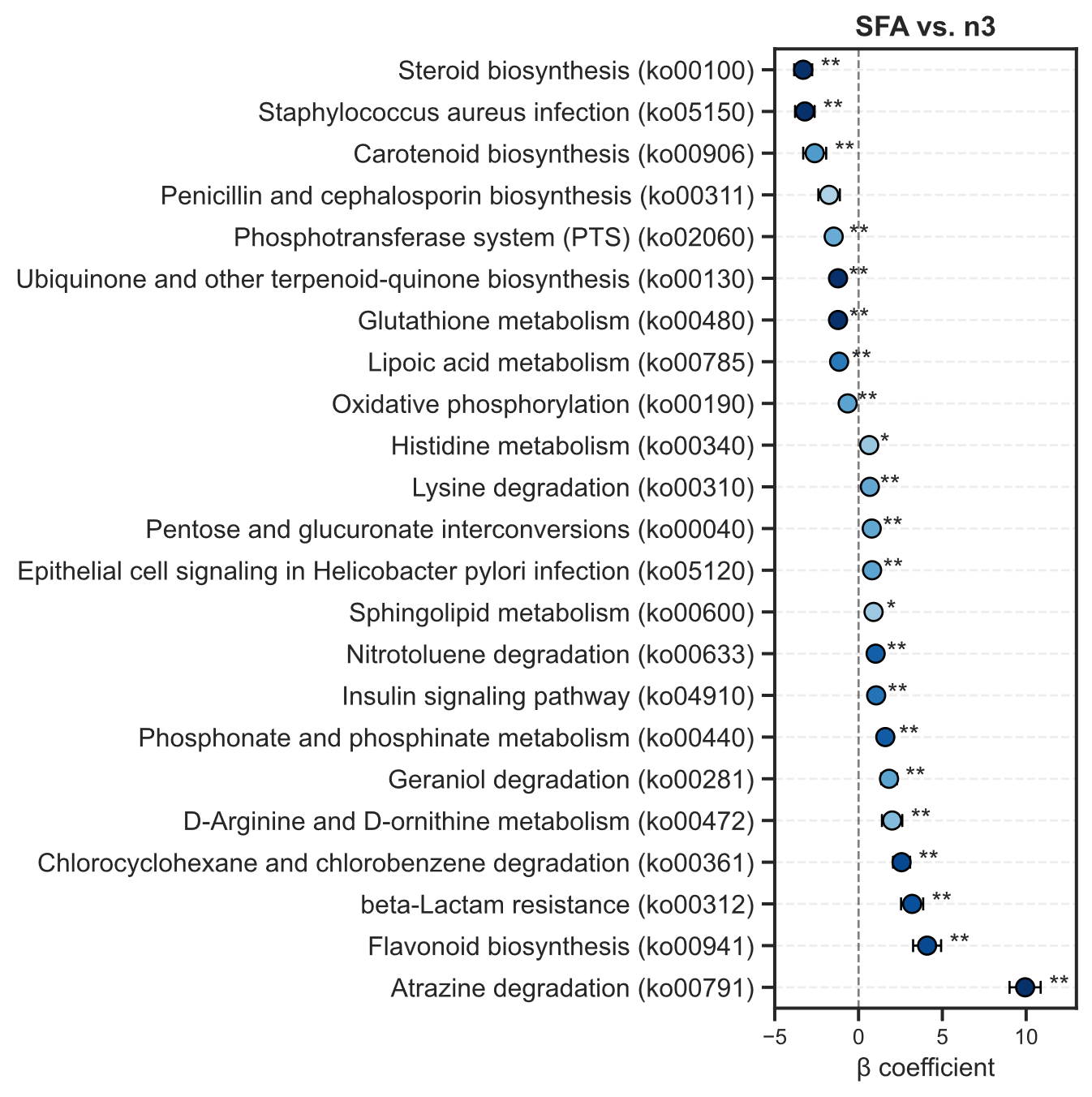


**
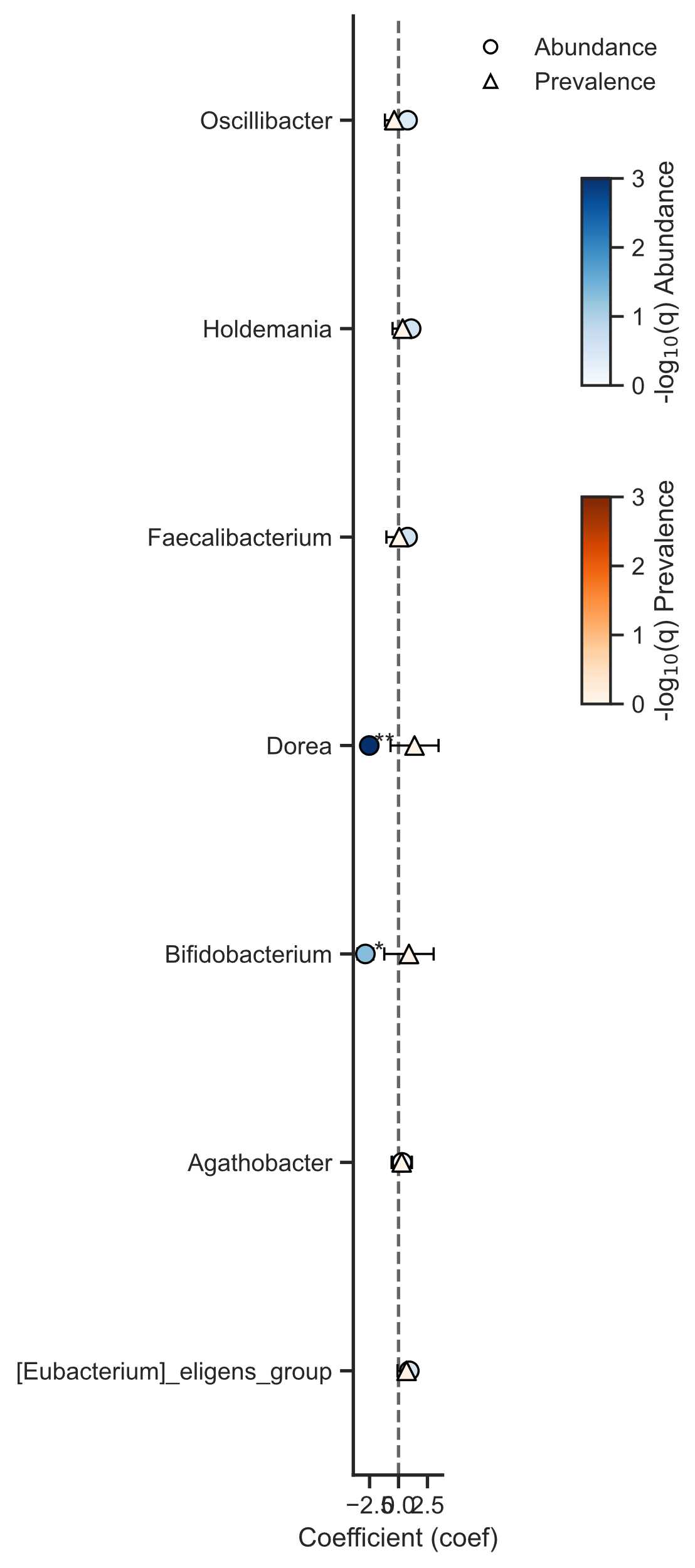
**

**Figure S3. Functional pathway prediction analysis based on PICRUSt2.** Multiple hypothesis testing correction was performed using the Benjamini-Hochberg false discovery rate (FDR), and adjusted q-values were reported. * q<0.1; ** q<0.05.


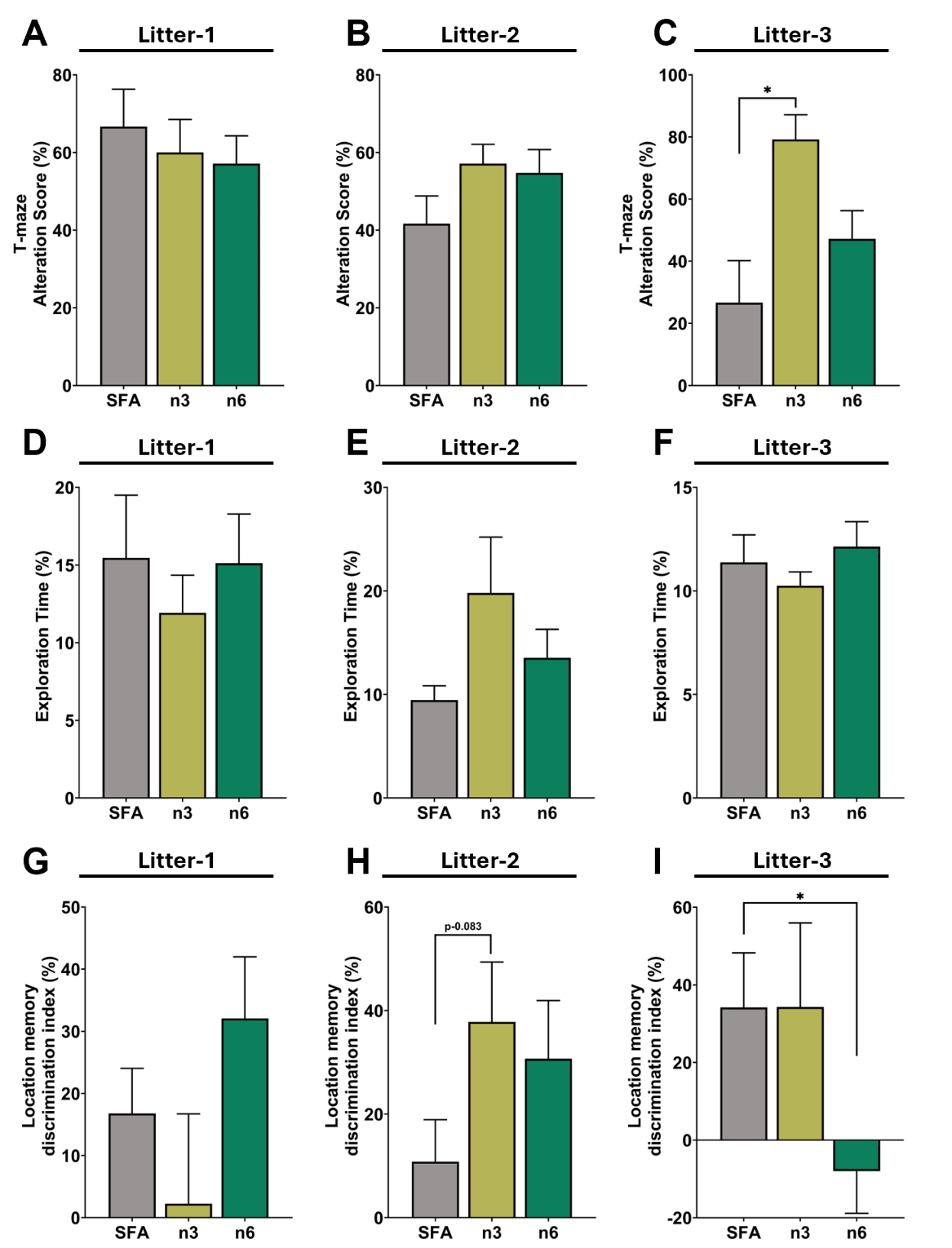


**Figure S4. Litter-wise comparison of neurobehavioral assessment. (A-C)** T-maze alteration score (%). **(D-F)** Exploration time (%). **(G-I)** Location memory discrimination index (%). One-way ANOVA with Tukey’s posthoc correction is used for neurobehavioral tests. Sample size litter wise: L1=5-7, L2=6-7, L3=4-6 * *p* < 0.05 between SFA and PUFA groups. Data are presented as mean ± SEM; n = 5-7 mice/group.


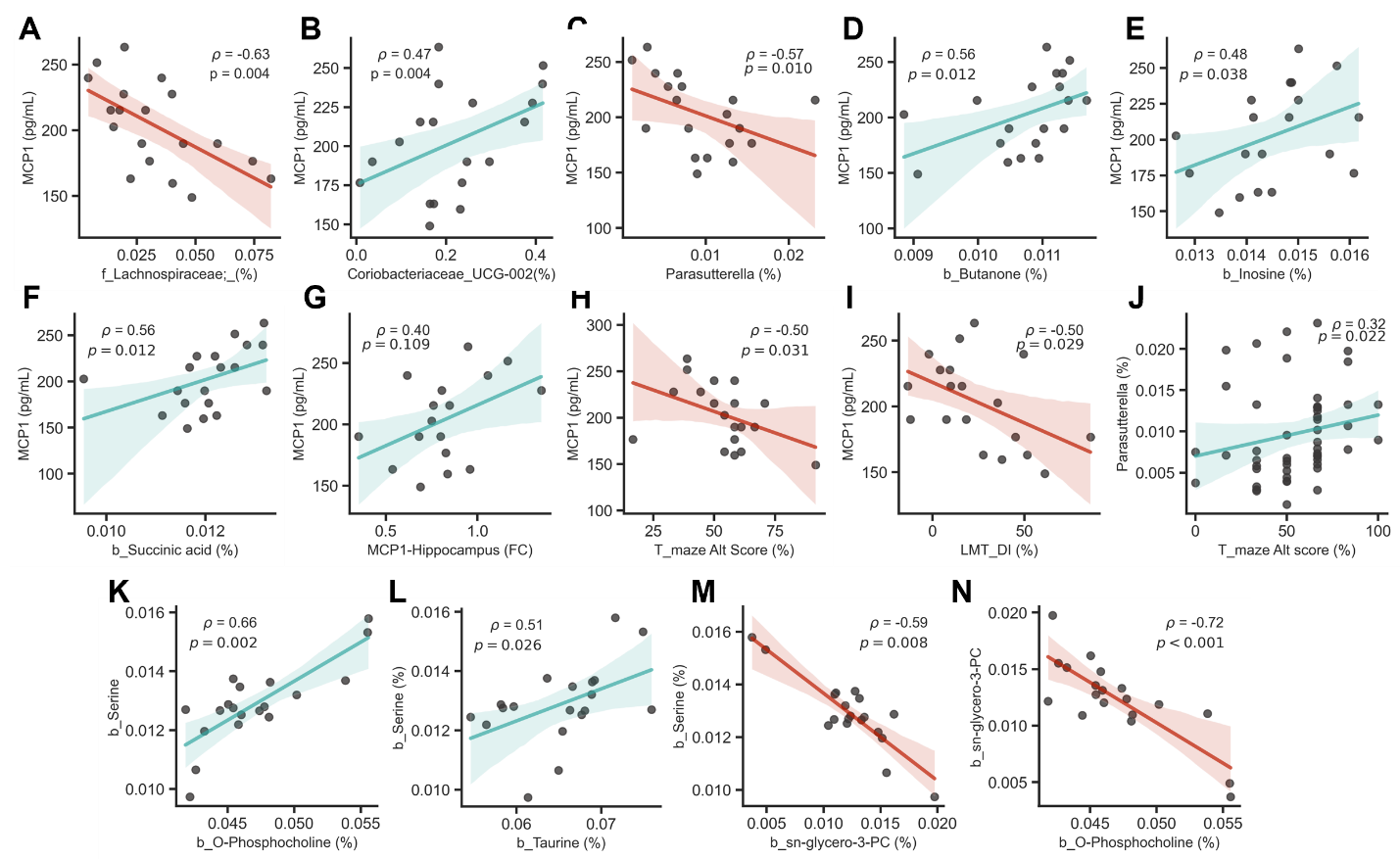


**Figure S5. Spearman correlation analyses. (A-I)** Association of MCP1 with key microbial taxa, brain metabolites and neurobehavioral tests. **(J)** Association of key microbial taxa with neurobehavioral tests. **(K-N)** Association between key brain metabolites.


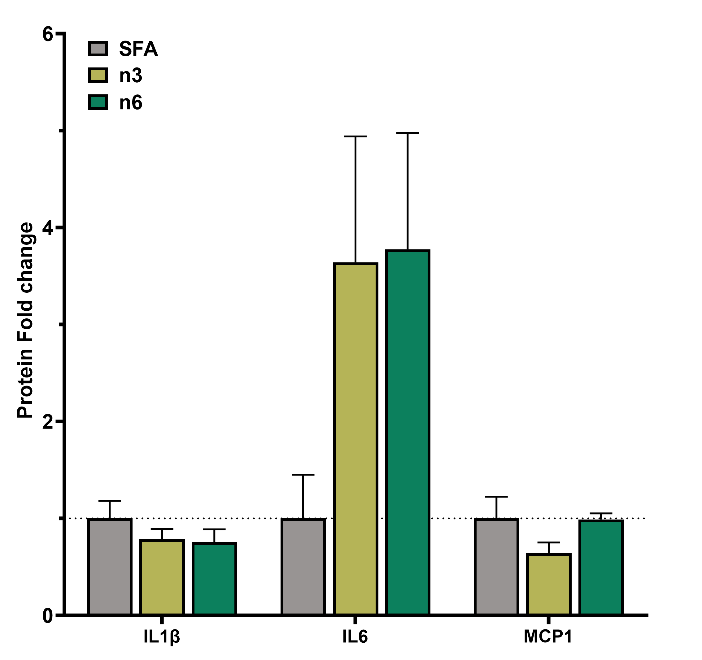


*

**Figure S6. Protein expression of inflammatory markers in the ileum of male mice.** Data are presented as mean ± SEM; n = 4-6 mice/group


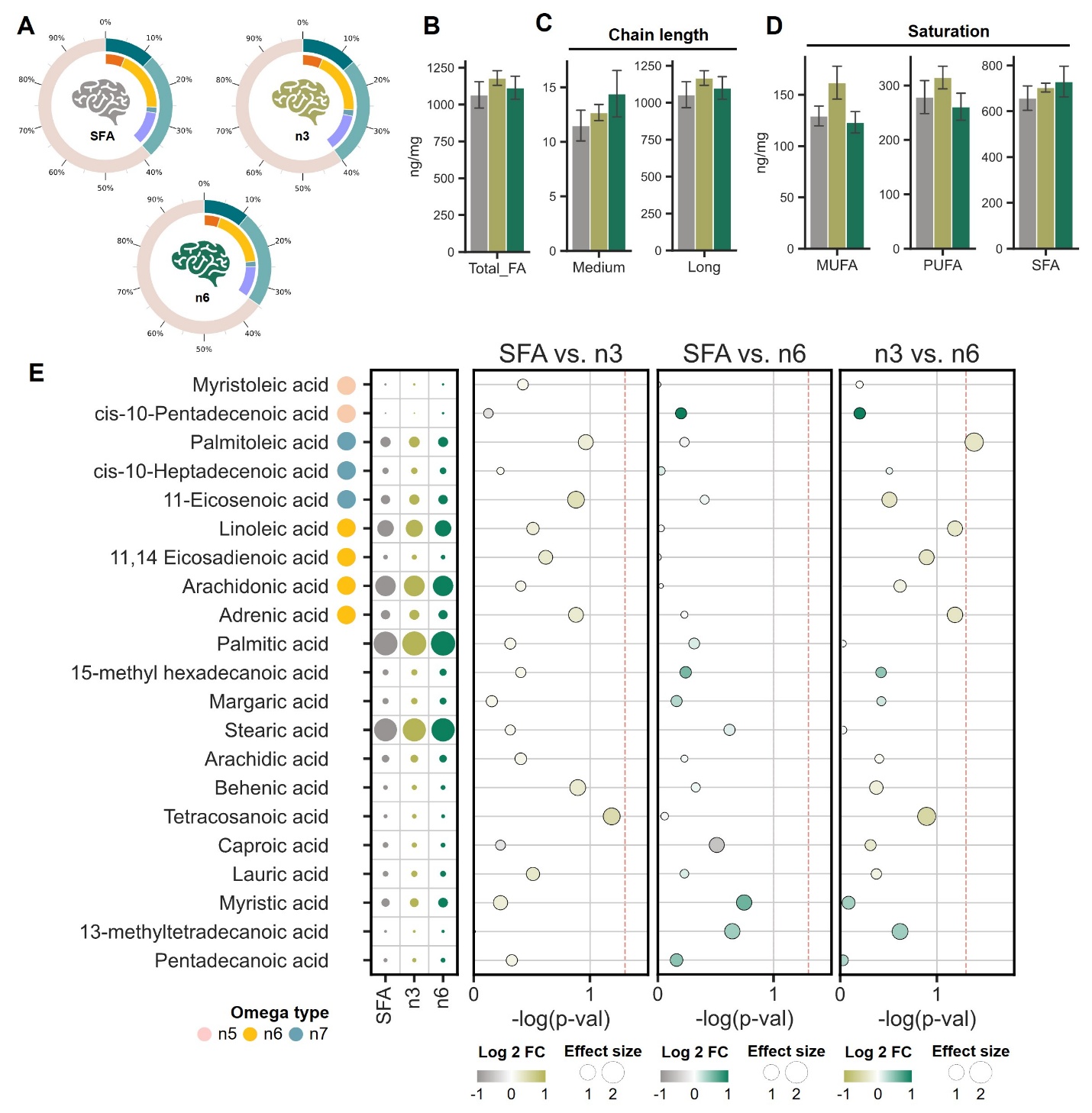


**Figure S7. Brain lipidomics. (A)** Relative proportion of fatty acids based on saturation and omega-types (%). **(B)** Abundance of total fatty acids (ng/mg). **(C)** Abundance of fatty acids based on chain length (ng/mg). **(D)** Abundance of fatty acids based on saturation (ng/mg). **(H)** Abundance and bubble plot of other brain lipidome data. The size of each bubble represents Cohen’s d effect size, and the color intensity indicates log2-fold change between the two groups. Significance (*p* < 0.05) is calculated using the Mann-Whitney U test and plotted as -log (p-value) for each metabolite. Data are presented as mean ± SD; n = 6 mice/group.
